# The expansion of colonial state healthcare in twentieth-century British Africa

**DOI:** 10.1017/mdh.2024.45

**Published:** 2025-01

**Authors:** Jutta Bolt, Jeanne Cilliers

**Affiliations:** ^1^University of Groningen, Netherlands; ^2^Lund University, Sweden

**Keywords:** public healthcare, colonial africa, public goods, Medical History, colonial government, health investments

## Abstract

We chart and assess the scope and utilisation of state-supplied hospital infrastructure in British Africa, c. 1900–60. Using archival sources, we examine the heterogeneity in colonial administrations’ investment into curative healthcare provision across various regions of British Africa. Our research highlights significant disparities in healthcare provision during the colonial period. These disparities were shaped by a range of observable factors, including differences in colonial policies, budgets, investment priorities, and the availability of medical personnel. We test stylised facts about public goods provision derived from previous literature and highlight the importance of understanding the historical context in shaping healthcare systems in Africa today.

## Introduction

After independence, most former British African colonies inherited health care systems that were established during the colonial period: a medical legacy that was largely curative rather than preventive.^1^ While many countries have since made significant efforts to expand and improve public health systems to better serve the needs of their populations, lack of funding and infrastructure, as well as a shortage of trained health workers, remain obstacles. The historical roots of these low health-related investments in Africa have long been recognised^2^ but the differential spread of healthcare across the continent has received less attention.

There is a longstanding, yet largely unresolved, debate that cuts across the various disciplines of history, economic history, and political economy, asking why health infrastructure was not established on a larger scale on the African continent under colonial rule. One critical demand-side factor was the resilience of local healing systems and indigenous perspectives on health, which both shaped and limited the reach of European medical institutions and practitioners. These local systems were deeply ingrained and more trusted by indigenous populations than unfamiliar European medical practices.^3^ On the supply side, some argue that the disease environment resulted in high mortality rates for Europeans in Africa, discouraging large-scale European settlement in favour of establishing extractive institutions to transfer the resources of the colony to the coloniser, and as a result, public goods provision was neglected.^4^ Others suggest that geographical conditions and foreign disease environments complicated the colonial administration of large territories resulting in ‘night watchmen’ states which financed minimal tasks at minimal cost.^5^

Both the extractive and minimalist perspectives assert that little was spent on development, even if for different reasons. Colonial healthcare systems were generally set up to serve the needs of European colonisers and at least initially, did not prioritise developing a public health system that would serve the needs of the indigenous population. In many cases, traditional healthcare practices and beliefs were ignored or actively discouraged.^6^ For the indigenous population, the colonial policy of health care provision was inadequate, underfunded, and understaffed.

At the same time, colonial investments in healthcare were not negligible. Frankema estimates that in 1925, an average of nine per cent of the budget was spent on healthcare in British Africa.^7^ Developments in public health and the ensuing reduction in the fatality of tropical diseases during the second half of the colonial period are thought of as some of the few tangible positive contributions of colonial rule to African development.^8^ The rudiments of these medical services date back to the nineteenth century, especially in West Africa, but it was not until the beginning of the twentieth century that the colonial administrations made progress in implementing more systematised, state-driven, and comprehensive medical policies, albeit non-uniformly across the continent.^9^ ^,^^10^

Our study sheds light on the heterogeneous scope and effectiveness of public health expansion in British Africa and is driven by two overarching research questions: To what extent was the expansion of public health services differentiated across former British Africa? And what explains public health systems’ performance relative to capacity? Policies and practices varied greatly among different colonies and were influenced by factors such as the economic and political situation in the colony, the priorities and policies of individual colonial administrators, and importantly, differences in local perceptions and adaptation of colonial healthcare measures.^11^

Using Colonial Annual Medical Reports, Colonial Blue Books, Colonial Annual Reports, and statistical abstracts, we provide comparable empirical evidence of the establishment and operation of state-supplied public health systems in twelve former British colonies for the period of 1900–60.^12^ Exploring the heterogeneity in British colonial public health provision while testing several stylised facts derived from previous literature about said provision is our main objective.

## Background and previous research

Throughout its pre-colonial history, various diseases spread across Africa via the trade and slaving routes connecting it with the Middle East, Asia, the Americas, and Europe. The continent also grappled with an array of endemic diseases, the combination of which took a significant toll on human life. Prolonged exposure to global forces over several centuries arguably brought some measure of immunity against imported infectious diseases.^13^ Many pre-colonial societies had specific natural and cultural mechanisms in place to control the incidence and severity of local diseases.^14^ For example, in Southern and East Africa during the mid-nineteenth century, societies prevented the spread of sleeping sickness by managing the habitat of the tsetse fly through controlled bush burning.^15^ In communities with a tradition of long-distance trade, such as those in West Africa, well-defined seasonal routes constrained transit traffic and contact between foreign traders and the local communities living off those routes, reducing the possibility that diseases would spread over larger areas.^16^

The disease equilibrium rapidly changed when Europeans intensified their commercial and military presence on the continent during the second half of the nineteenth century. The colonial administration expanded its trade networks to facilitate the transportation of people and goods necessary for the economic exploitation of newly acquired territories, creating new transmission pathways for infectious diseases.^17^ Forced labour institutions disrupted existing settlement patterns, and the high demand for unskilled labour for, among others, cash crop production on plantations, mining, and construction increased migrant labour flows moving people into unfamiliar disease environments. At the same time, the focus on increasing cash crop production and mining output diminished food production, increasing the incidence of famine and malnutrition, and leading to deteriorating health outcomes for the affected populations.^18^ Rural populations were increasingly compelled to live in larger villages or alongside trade routes. With rising urbanisation came overcrowding and inadequate water and sanitation services, progressively turning cities into hubs of disease.^19^ Recurrent and devastating epidemics characterized the nineteenth and early twentieth centuries, negatively affecting population developments, especially in Eastern Africa.^20^

While colonial rule undeniably transformed the health landscape of Africans by altering the continent’s disease environment, Europeans were also responsible for the introduction of modern preventive and curative medicine to the continent, though these efforts were far from a singular cohesive force. African societies had a long tradition of local healing systems, although typically small-scale and personalized.^21^ In contrast, colonial healthcare initiatives, while starting localized and often small-scale formed the basis of subsequent state-level public health service provision.^22^ Greenwood highlights that colonial medical officers frequently collaborated with various groups, sometimes even those whose objectives diverged from those of the British government.^23^ This interplay is evident in the role of medical missionaries, who from the mid-nineteenth century embedded themselves within local communities, blending their evangelical missions with healthcare provision.^24^ Outside major administrative hubs, Western medical care largely depended on mission efforts until the eve of the First World War. However, in larger cities such as Lagos, Nigeria, where the first colonial medical post was established in 1862 and the first colonial military hospital in 1871, or in the Gold Coast, where the Colonial Medical Department was created in 1880, significant advancements were already made in colonial medical infrastructure.^25^

More institutionalised healthcare was established piecemeal in the various British African colonies from the last decades of the nineteenth century onward. For example, in Nyasaland, the first medical officer was appointed in 1891 while the formal establishment of the Medical Department followed five years later, in 1896. In Uganda and Kenya, the Medical Department was established at the beginning of the twentieth century, while the colonial government established a more formal healthcare system in Northern and Southern Rhodesia only when it took over from the British South African Company (henceforth BSAC) in the early 1920s.^26^ In some places, the central colonial government was to various extents ‘assisted by the active cooperation of the native authorities (local governments), who themselves in many cases finance medical and health services in their areas’.^27^ Finally, there were a small number of private medical practitioners and other voluntary agencies (both of international and local character) engaged in healthcare provision.^28^

The impacts of the various local and foreign health service providers have already been studied for several individual cases, for example, Malawi,^29^ Tanganyika,^30^ the Gold Coast,^31^ Southern Rhodesia,^32^ Uganda,^33^ and Sudan.^34^ On the evolution of the respective colonial healthcare systems, several key themes emerge including the inadequate level of finance, staff shortages and, healthcare provision segregation between Europeans and Africans particularly in the early stages of healthcare services, with Patterson noting increasing acceptance and demand for Western biomedicine in colonial Ghana only after the 1930s. Regional studies provide insights into both the West and East African experience.^35^ Beck considers the role of colonial governments in healthcare provision in East Africa between 1900–50, showing how medical policies were affected by the prevailing trends in the political interpretation of the duties of the colonial power resulting in changing medical services through the colonial period.^36^ Nkwam, studying the health services in British West Africa between 1920 and independence, provides a detailed account of the shift of emphasis from a medical policy geared to treating expatriates towards more care for the African population and demonstrates how this was hindered by continuing staff shortages.^37^ Several studies focus on British West Africa during the first decades of colonial rule. Gale discusses the colonial government’s response to the various problems the colonial medical service faced during the initial phase of establishment, while Johnson describes structural barriers and the self-interest and conservatism of medical officers, which limited the implementation of even the most basic public health measures in West Africa.^38^ Crozier investigates the role of medical practitioners in shaping the colonial identity in British East Africa^39^ and, in a collection of essays, extends this investigation ‘beyond the colonial state’ to complementary providers of health care.^40^

Another area of literature examines colonial medicine and medical interventions within a broader context. Lee, for instance, highlights the diverse landscape of healthcare development across the continent, emphasizing the contributions of various actors, including indigenous societies and healing systems, missionaries, colonial and post-independence governments, and international organizations.^41^ Giles-Vernick, James, and Webb^42^ delve into the history of global health initiatives, focusing on significant health interventions during colonial rule and their implications for contemporary practices. Feierman and Janzen investigate the social, political, and economic contexts of health, disease, and healthcare practices and explore how ideas about disease transmission influenced urban planning in colonial African cities.^43^

While informative, these studies only facilitate limited comparisons across time and space due to, for example, differences in their aims and scope, the incommensurate periods covered, and the sources and indicators assessed. In this paper, we focus specifically on healthcare services provided by the central colonial government, which over time, organised its healthcare provision into a medical branch that dealt with the curative side of medicine, and a health branch that dealt with the preventive side.

## State-supplied health care in British Africa

The medical department was the second biggest personnel branch of the British Empire, with the Colonial Medical Service employees making up nearly a third of all Colonial Service staff. The initial aim of colonial healthcare provision throughout British Africa was the protection and improvement of European health.^44^ Throughout the colonial period, but acutely in the early years, insufficient financial resources and a lack of trained personnel were a constant source of frustration for medical officers who often complained of not having basic medical equipment. Due to chronic understaffing, it was common for medical officers to be otherwise engaged in non-medical duties: securing senior posts for the advantage of gaining lucrative but competitive private practices in the region or serving as interim district councillor when the incumbent was on leave.

The colonial system of mostly urban hospitals was, as in the case of India, initially confined to serving members of the colonial establishment. The first decade of the twentieth century, however, was punctuated by devastating epidemic episodes of sleeping sickness (among the most notable in Uganda starting around the turn of the twentieth century),^45^ news of which ostensibly permeated British consciousness to slowly produce a new attitude towards extending health services to Africans.

The rejection of numerous potential military recruits due to their poor health reiterated the need, in the immediate aftermath of the First World War, for colonial authorities to increase their efforts in addressing preventable health conditions of local populations.^46^ A further series of epidemics in Nigeria, widespread outbreaks of yellow fever throughout West Africa in 1926–27, and an increasing awareness of the welfare of colonised peoples’ need for greater medical and health services (not least so that African labour could be productive and pay taxes, and so that the general population could take up manufactured goods from the UK).^47^ ^,^^48^ The period that followed saw everywhere a considerable expansion in state health services for Africans, now including outpatient clinics and rural dispensaries. It reflected the change in emphasis of colonial policies after the First World War, which was formalized by the adoption of the Colonial Development Act (hereafter CDA) in 1929, based on the idea of broader state responsibilities with regard to colonial social and economic development that had already been around since the Colonial Loan Act of 1899.^49^ One of the most important policy shifts during the 1920s was directed towards mass disease eradication campaigns against smallpox and yaws.^50^

By this time, owing to advances in scientific knowledge and awareness of the local health conditions, slow but real progress became possible.^51^ Increasingly both European and newly trained African medical personnel provided basic forms of healthcare, although personnel shortages and the quality of training for local staff remained bottlenecks to the broader provision of medical services and to hospitals catering for African patients.^52^ African trust in Western medicine was gradually built as medical services improved and their benefits became more visible.^53^ This did not mean that local healing practices were abandoned. Most patients still primarily visited local healers, incorporating some biomedical treatments into their broader healing practices.^54^

The substantial progress made during the 1920s, especially in the area of curative healthcare, came to an abrupt halt in the early 1930s due to the Great Depression and its effect on government finances. Medical expenditures fell, projects were abandoned, vacant posts were not filled, and maintenance costs were cut.^55^ In the aftermath of the Great Depression colonial governments, imbued with a new sense of purpose and financing, refocused on expanding healthcare services as the CDA of 1929 authorised expenditure on colonial development projects including the promotion of public health. However, progress in this regard was uneven. On the one hand, the colonial government in Nigeria used these funds to invest in, inter alia, dispensaries (largely run by Native Authorities), maternity and sanitary inspector training centres, the provision of mobile ambulances, and sanitary improvement schemes.^56^ On the other hand, the colonial governments in the Gold Coast, Sierra Leone, and The Gambia used funds from the CDF largely to improve infrastructure.^57^ A more uniform trend emerged in interwar East Africa.^58^ Although government expenditures rose throughout East Africa until 1931, funds were more restrictive for expansionary medical ambitions, particularly in Tanganyika with its geographically dispersed health locations. Additionally, the effects of the recession were felt longer in East versus West Africa, lingering until 1937.

Against the backdrop of the Great Depression, the early 1930s stands out as a watershed period as it saw colonial medical authorities and representatives of international health organisations collaborating locally and internationally, in consultation with various government departments with a broader vision of public health.^59^ This was a continuation of a broader intellectual movement beginning in the 1920s in Europe towards a more holistic understanding of how social and economic conditions affect health and disease.^60^ Colonial healthcare policy underwent a shift from an emphasis on controlling the disease environment and providing curative care, towards a policy that sought to change healthcare outcomes more through prevention and behaviour modifications.^61^ Despite a prevailing consensus that a broad-based approach to health rested on education and economic improvement, many military-style disease-focused campaigns existed during this period.

In light of the growing awareness of the poor indigenous health conditions in many colonies, and to counter the influence of anti-colonial movements led by an expanding urbanised African elite within Africa, the founding of the United Nations (UN), and the emergence of a new world order internationally, colonial governments officially launched a broader development agenda. The Colonial Development and Welfare Act (1940) increased funding for public health programmes and the training of indigenous medical staff and focused on improving the health of the indigenous population.^62^ The shift towards more development-oriented expenditure aimed to provide the benefits of modern medicine to the largest population possible and included a shift away from capital to recurrent expenditure, focussing on for example, salaries, maintenance and administration.^63^ However, both financial stringency and the inadequacy in training and employing African medical staff continued to handicap hospital expansion throughout British colonial Africa.

## Analytical framework and hypotheses

**Figure 1.**
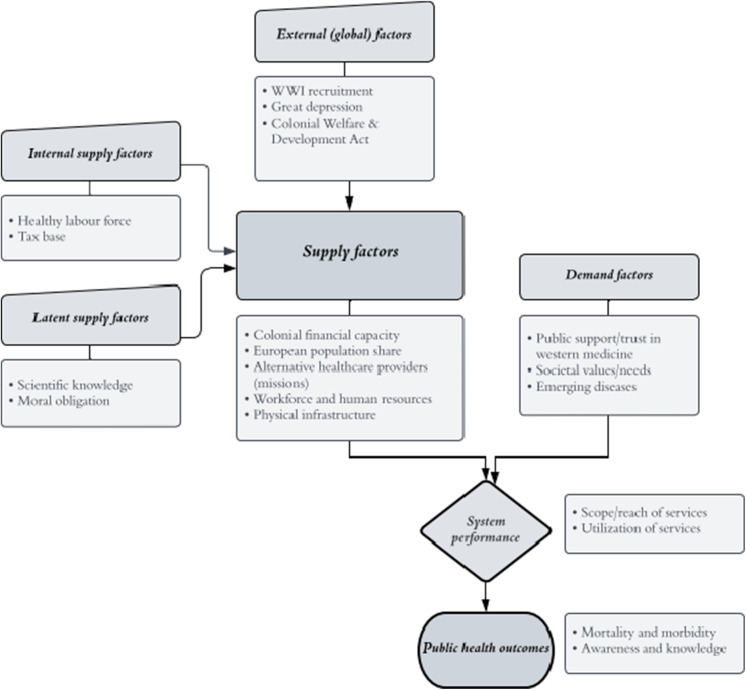
Analytical framework, inspired by Meyer, Davis and Mays. *Note:* Anne-Marie Meyer, Meredith Davis, Glen P. Mays, Defining Organizational Capacity for Public Health Services and Systems Research, *Journal of Public Health Management and Practice*, 18, 6 (2012), 535–44.

Much of the contemporary public health literature focuses on public spending on health and its link to population health outcomes, typically measured by mortality or morbidity rates or the level of societal awareness and knowledge about good health and hygiene practices.^64^ Since we do not observe population health outcomes, the focus of our study is limited to examining the relationship between, broadly speaking ‘capacity’ and ‘system performance’. We aim to understand which factors are associated with system performance, which we conceptualise as both the *scope* of public health care services, approximated by the number of state hospital beds per capita for African patients and the number of state hospitals per capita^65^, and the *utilisation* thereof, measured by the number of in- and outpatients per capita. Both scope and utilisation are in turn influenced by various supply and demand factors. In this section, we draw on existing literature on the effects of British colonialism on health services to develop a framework and form hypotheses about the association between capacity and the provision and uptake of public health services during the colonial period (see Figure 1).

On the supply side, the two most stringent and direct constraints on colonial state capacity to provide public health services throughout the colonial period were the size of the annual budget and the availability of medical staff.^66^ Due to the British colonial policy of self-sufficiency, funds for administrating colonial territories needed to be raised within the colonies.^67^ This meant that colonies with small revenue streams had fewer resources at their disposal to invest in public goods provision. It is not uncommon to find occurrences in the Annual Medical Reports of Chief Medical Officers lamenting the fact that while there was a strong willingness to expand medical services, the dire state of medical department finances left little room for improvement. In Nyasaland, for example, the 1928 annual medical report noted that rural dispensaries, typically staffed by local dispensers and occasionally assisted by unskilled aides, were in high demand, with repeated requests from the District Commissioner to increase their number. Expansion, however, was constrained by limited funds for construction and maintenance, and insufficient training facilities for dispensers.^68^ Given the stringency of the budget across countries and time, we expect a positive association between colonial budgets (or the share of the budget allocated to the medical department) and system performance.

Difficulties in attracting medical personnel to staff the healthcare service was the second direct constraint to expansion that all colonies faced. In the early years of the twentieth century, few Africans had formal medical education, and in West Africa, trained African physicians were actively barred from accessing higher posts in the medical service^69^, despite most territories in Africa holding little appeal for the majority of Britain’s medical graduates.^70^ The Colonial Medical service was not considered an attractive career: payment was often not competitive compared with private or domestic government practice and conditions in the colonies were known to be harsh with limited facilities.^71^ As a result, colonial medical departments were chronically understaffed with acute shortages during the inter-war period, particularly in the early 1930s, as the government was compelled to reduce the number of medical officers due to the economic crisis.^72^

With increasing medical ambitions after the First World War came a more general understanding of the importance of training African medical staff in order to scale up medical outreach. In 1919, Dr Wiggins, head of Uganda’s medical department wrote that the most pressing issue was the foundation of a government-operated medical school to train local medical staff.^73^ Similarly in West Africa, colonial authorities quickly realised that it would be impossible to recruit or pay for enough British doctors to provide mass healthcare. While the employment of African medical personnel was deemed to be essential for a functioning public health system, the training of African health workers lagged far behind in British West Africa compared with, for example, French West Africa.^74^ Technical education for Africans in the former only began during the 1920s and was to a large extent provided by missionaries.^75^ This training however offered little advanced education for developing skills beyond nurses, dressers, and dispensers. Therefore, despite a slow Africanisation of the medical staff in British colonial Africa, human resources remained an important direct supply constraint affecting system performance.^76^ We expect a positive association between the number of trained European medical staff (relative to the size of the population) and system performance.

Beyond financial capacity and the scarcity of trained medical personnel, the literature on public goods provision in Africa suggests four other important factors affecting healthcare provision: regional variation, European settler presence, missionary intensity, and global factors, each of which will be discussed in more detail below.

### Regional variation

Regional economic differences theoretically place West Africa in a position to perform better than East or Southern Africa, partly due to its more rapid economic expansion generating larger government budgets.^77^ This we capture in our discussion of financial capacity above and control for directly in the model. West Africa was also the region with the longest exposure to European presence and colonial rule. The British colonial medical service started first in West Africa and as a result, this region had longer experience in building up medical bureaucratic capacity and experience in providing healthcare, and was the region exposed longer to Western medicine.^78^ We control for this directly by including a variable capturing the years since the establishment of the medical department in each colony.

Greater emphasis was placed in West African colonies on reducing high European mortality rates, potentially stimulating greater investments in the medical department. Furthermore, already in 1902, the West African Medical Staff (WAMS) brought together the six medical departments of British West Africa^79^ intending to enhance the recruitment of European medical officers for service in the region,^80^ offering a pay supplement to compensate officers for living in the challenging climates of the Gold Coast, Nigeria, Sierra Leone or The Gambia.^81^ Within Sub-Saharan Africa (SSA), the WAMS was considered most the prestigious posting. This might have attracted the best medical officers to this region.

In contrast, in East Africa, medical departments were slower to develop. From the end of the nineteenth century, new state-controlled medical administrations slowly developed in both Kenya and Uganda. Apart from a brief period between 1903–8, the two medical departments remained independent, and the expansion of service provision remained limited as medical departments were not generally prioritised.^82^ After the First World War, Tanganyika was added to British East Africa, and in 1921 the East African Medical Service (EAMS) formally united the medical services of Kenya, Uganda, and Tanganyika in order to standardise pay scales and regulate terms and conditions of employment throughout the regions. While the EAMS trickle-down was comparatively less prestigious than the WAMS it was still considered preferable employment to the very small medical services of Nyasaland and Northern and Southern Rhodesia.^83^ As a result, we expect to find that West African colonies invested more in health infrastructures (larger scope in terms of the number of state hospital beds for Africans per African capita and state hospitals per capita) and reached more patients (utilisation) throughout the colonial period compared with both East African and Southern African colonies.

### Settler presence

The establishment of public health care services in all of British Africa was at least initially, aimed at protecting and improving European health.^84^ Until 1920, the system was designed to care only for government employees. As a result, the initial locations of government health centres and staff were determined by where European officials and other settlers resided – mostly in urban areas – and not necessarily by African population density.^85^ Moreover, especially in colonies with a larger proportion of expatriates in the population, settlers often represented an important economic and political force.^86^ In these colonies, there was stronger racial segregation and a continuation of the focus on improving European healthcare provision. As a result, we expect fewer, mostly urban, hospitals geared towards healthcare for Europeans, in settler colonies – hence a negative association between the proportion of Europeans in the total population and the number of hospitals per capita for Africans (scope). Additionally, we expect that these hospitals provided less care for Africans due to stronger racial segregation and more pronounced antagonism towards welfare provision for Africans.^87^

### Missionaries

The next important factor for state healthcare provision in British Africa was the presence of alternative or competing providers. Missionary societies often spearheaded medical service provision from the mid-nineteenth century onward, and the bulk of medical care prior to the First World War, especially in rural areas, was in the hands of missions.^88^ Missionaries were also important in swaying public opinion in favour of European-provided healthcare services. Medical missions were often welcomed even in situations where Africans remained hostile to their presence during the early years of contact. These missionary societies set up clinics that offered basic primary medical care in rural areas such as dispensing medicine, dressing wounds, and diagnosing minor ailments. Some also built hospitals that provided more sophisticated medical services such as specialist or surgical care for Africans in the more densely populated areas.^89^

Missionaries were generally acknowledged as helpful to the colonial government, although the relationship between the two was often complicated. Missionaries had better contact with local African populations and were therefore in a better position to convince Africans to accept the Western treatment of disease. Before the First World War, colonial officials had generally been content to subsidise missionary societies to take care of medical services in rural communities. This changed after the war as colonial governments became increasingly aware that providing medical services was an important way of establishing contact and influence with local populations. The lack of medical standards in medical missions and increasing fears that medical missionaries might act contrary to government ideas on the treatment of Africans, made colonial governments more reluctant to grant permanent subsidies to medical missions. During the 1930s, the expansion of government medical work appeared to have led to the reduction of medical work done by missions. However, the practical need for cooperation remained since colonial territories were vast, and colonial medical departments were chronically underfunded and understaffed.^90^ Resources and expertise were frequently pooled, both formally and informally, between the colonial medical service and other local health agencies.^91^ After the Second World War, the distinction between missionary and secular colonial medical services became less distinct. The medical efforts of missionary societies became gradually secularised and increasingly absorbed into the state.^92^ The association between the number of mission health providers and system performance both in terms of scope and utilisation could therefore vary across space and over time, but we expect that the initial collaboration between state and non-state actors outweighed later tensions.

### Global shifts

The final group of factors were global/external shifts, affecting both policy orientation and financial capacities, such as the colonial development welfare programmes and the global recession of the 1930s. We aim to capture these shifts in policy and financial capacity by distinguishing three sub-periods. The first is from 1900–20, which serves as the baseline period characterised by relatively limited progress in healthcare provision due to both the predominant European focus of medical policy and limited financial capacity. The second period ranging from 1920–40 saw a considerable expansion of healthcare during the 1920s and at the end of the 1930s, reflecting the change in emphasis of colonial policies after the First World War, both based on the idea of broader state responsibility with regard to colonial social and economic development, and out of self-interest so that African labour could be productive and pay taxes to the colonial state. Overall progress during these decades was curtailed however by the Great Depression of the early 1930s as it depressed the financial capacity of colonial governments to invest. During the last period, 1940–60, colonial governments launched a broader development agenda resulting in a substantial increase in expenditure in social development, including healthcare. To carry out colonial development and welfare programmes, colonial states began to receive significant subsidies (grants-in-aid) from the metropole after the Second World War.^93^ In practice, this meant the end of the self-sufficiency policy for the colonies. However, financial means were often still not sufficient to meet ambitions.

Finally, on the demand side, trust in and acceptance of (certain forms of) biomedicine influenced the degree to which Africans up colonial state-supplied healthcare services. During the initial years under colonial rule, colonial administrators attempted to engage local leaders to persuade their communities of the benefits of taking up these services. In Bechuanaland, for example, it was reported that ‘[the] Acting Chief at Mochudi [had] been of great assistance to the Medical Officer there in encouraging his people to attend the dispensary instead of consulting witchdoctors, and his example [would], doubtless, be a stimulus to others in the same direction’.^94^ Furthermore, it was reported that ‘the local Chief, who exercises great influence, is to a great extent the determining factor by his advice and encouragement to his people to go to the Medical Officer for medical aid, or not’.^95^ Unfortunately, we lack consistent information on the rate of acceptance, or of local demand for colonial healthcare and we therefore do not include this in our analysis.^96^

From the contextualisation and analytical framework discussed above, we form the following testable hypotheses about the association between capacity and the provision and uptake of public health services:

H1: The scope and utilisation of public health systems was higher in West African colonies resulting from trickle-down effects from larger investments in response to the harsh disease environments for Europeans and the greater presence of trained medical staff.

H2: The scope and utilisation of public health systems was lower in settler colonies resulting from a lower rural investment due to a larger urban European presence and stronger racial segregation in settler colonies.

H3: Colonial governments provided less healthcare (both scope and utilization) when more complementary healthcare providers were present.

H4: Colonial governments’ healthcare provision responded to external (global) factors.

## Sources and data

We trace the expansion of public health services in twelve British African colonies from 1900–60, through the creation of a new panel dataset containing total government revenue and expenditure, expenditure on medical departments, staffing, the number of hospitals, bedded dispensaries, and other charitable medical institutions, and the number of in- and outpatients receiving treatment at government hospitals. The countries studied are grouped into three broad medical administrative regions: 1) the West African Medical Service (WAMS) comprised of The Gambia, Sierra Leone, the Gold Coast, and Nigeria; 2) the East African Medial Service (EAMS), which formally united the services of Kenya, Uganda, and Tanganyika (we include Zanzibar here) in 1921; and 3) Southern Africa comprised of Bechuanaland, Nyasaland, Northern Rhodesia, and Southern Rhodesia.

Annual Medical Reports (AMRs) are the main source of primary data. These were obtained from the Governing Africa collection of the British Online Archive. The collection contains numerous documents sent to the British Government by its colonial administrations in Africa during the nineteenth and twentieth centuries. While the collection covers over a dozen disparate territories, researchers today can benefit from the fact that these annual reports contain a roughly consistent set of content, albeit idiosyncratically formatted at different points in time. All medical reports start with a section on administration, which includes a subsection on staff and another on finance. This is followed by a general description of the health situation in the colony, followed by a section on vital statistics mostly for the expatriate population, and a detailed overview of the annual medical budget (revenues and expenditure). Then, various sections follow that contain both descriptions and statistics on, for example, the general epidemiological state of the colony, general and specialised hospital services provided, sanitation services provided, the number of treatments per disease, and cause-specific mortality estimates. Most medical reports contain a statistical appendix including tables with returns on different matters such as the number and the location of hospitals and other healthcare facilities, the number of patients treated at various facilities, and the location and description of sanitation works being carried out.

Defining what constitutes a ‘hospital’ during the colonial period is complex. One of the primary distinguishing features of a hospital as recognized by the colonial administration was its ability to offer inpatient care, with designated wards for overnight patient stays, contrasting with rural clinics which provided outpatient services. These hospitals were equipped with slightly more advanced medical equipment and were generally staffed by at least one trained medical professional, such as a doctor or nurse, allowing them to treat more serious illnesses and sometimes carry out surgical procedures. However, there was a clear racial disparity in access to this healthcare; Europeans often received superior care, while Africans typically faced minimal and discriminatory treatment. The variability in hospital size and resources reflected an urban bias, concentrating facilities in strategically important areas. Our analysis focuses on hospitals and inpatient beds per capita, specifically considering facilities accessible to African patients and excluding those exclusive to Europeans.

The Colonial Medical Reports, like many colonial records, while valuable, have significant limitations. Chiefly, these documents convey only the European perspective, embedding conceptual and narrative frameworks that often portray Africans as subjects of European actions and ideas.^97^ In our context, they carry limited suitability for understanding African experiences of disease and treatment. The purpose and the context in which these records were created also shape their representativeness. Colonial administrators were likely motivated by administrative, economic, or political interests potentially leading to a skewed representation of events and conditions often ignoring or misrepresenting local perspectives and experiences. For example, Rouanet argues that colonial administrators had an incentive to deliberately bias the reported health figures upwards to use as propaganda or to overstate the positive impact of colonial rule.^98^ ^,^^99^ In addition, the statistical capacity of colonial governments also varied by colony and over time, leading to potential underestimation, censoring and selection effects.

Particularly relevant for the current paper are issues related to the number of hospitals and the number of patients treated (see the method section below). Regarding the number and locations of colonial healthcare facilities, the AMRs indicate some inconsistencies in the classification of hospitals and dispensaries, however, fluctuations in our series are minor and infrequent. Reliability concerns with respect to patient data are of a different order. First, only persons who actually visited a colonial healthcare facility were recorded, leading to an underrepresentation of the total potential patient basis. Additionally, patient characteristics likely changed over time as healthcare services expanded to provide care for Africans; most patients were initially men and only later started to include African women and children.^100^ Furthermore, records of patient admissions might be unreliable, especially for so-called outpatients mostly treated at (rural) clinics or dispensaries. However, it is difficult to assess the extent of this bias in the data.^101^ Owing to the varying degree of reliability of colonial official documentation our data processing involves significant cross-validation. The Colonial Blue Books, Colonial Annual Reports, and Statistical Abstracts of various colonies serve this purpose wherever possible.

## Method

Stemming from our hypotheses we are interested in testing the association between various supply factors on the performance of public health systems over time. We model system performance in two ways: First, we consider the *scope of government hospital services*, proxied respectively, by the number of beds for African patients in state hospitals per capita, and the number of state hospitals per capita. Second, we consider the *utilisation of government hospital services*, proxied respectively by government hospital inpatients per capita, and outpatient attendances at government hospitals and dispensaries per capita.^102^

We estimate a series of panel regression models with the following specification:
(1)

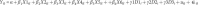

Where, in our first model of *scope*, *Y_it_* is the natural log of the number of beds for African patients in government hospitals per capita in country *i* at time *t.* In our second model of *scope, Y_it_* is the natural log of the number of government hospitals per capita in country *i* at time *t.*

In our first model of *utilisation*, *Y_it_* is the natural log of the number of government hospital inpatients per capita in country *i* at time *t.* In our second model of *utilisation*, *Y_it_* is the natural log of the number of out-patient attendances per capita at government hospitals and dispensaries in country *i* at time *t.*

*α* is the intercept. *X*1*_it_*, *X*2*_it_*, *X*3*_it_*, *X*4*_it_*, *X*5*_it_*, and *X*6*_it_* are the independent variables for country *i* at time *t* and include, respectively, log real total government revenue per capita as a measure of a country’s overall budget; log share of total government expenditure that is devoted to the medical department as a measure of the administration’s prioritisation of public health relative to other fiscal expenditures; log years since the establishment of the first medical department, to capture exposure to Western medical practices; log European medical staff per capita as a measure of human resources. For comparability, all nominal values were deflated using relevant price indices.^103^ ^,^^104^

In our first model of scope, we also include the log number of government hospitals while in the second model of scope, we substitute this for log average beds per government hospital as a measure of hospital size. In both models of utilisation, we include both of the latter as additional independent variables.

*D*1*_i_*, *D*2*_i_*, and *D*3*_i_* are dummy variables and *γ*1, *γ*2, and *γ*3 are their coefficients which relate to the hypothesis we set out to test. These are our main outcomes of interest, the first of which captures region by creating two dummy variables: one for East African colonies (Kenya, Uganda, Tanzania, Zanzibar) and one for Southern African Colonies (Botswana, Malawi, Zambia, Zimbabwe). If a colony is West African (The Gambia, Ghana, Nigeria, Sierra Leone), both dummy variables are 0 (hypothesis 1). A second dummy variable is included which takes the value of 0 when a country is defined as being a non-settler colony and 1 when a country is defined as being a settler colony (hypothesis 2). We follow the settler non-settler distinction found in the literature.^105^ ^,^^106^ Hypothesis 3 is tested via the inclusion of the log of the number of mission hospitals in each colony as a measure of non-state healthcare alternatives. Finally, a categorical variable for the period is created for 1900–19; 1920–39; and 1940–60 to capture the effects of external (global) events (the First World War, the interwar period, the Colonial Development Acts), on investment into public health systems (hypothesis 4). *u_it_* is the country-specific random effect and *ϵ_it_* is the error term.

## Results: General trends

**Figure 2.**
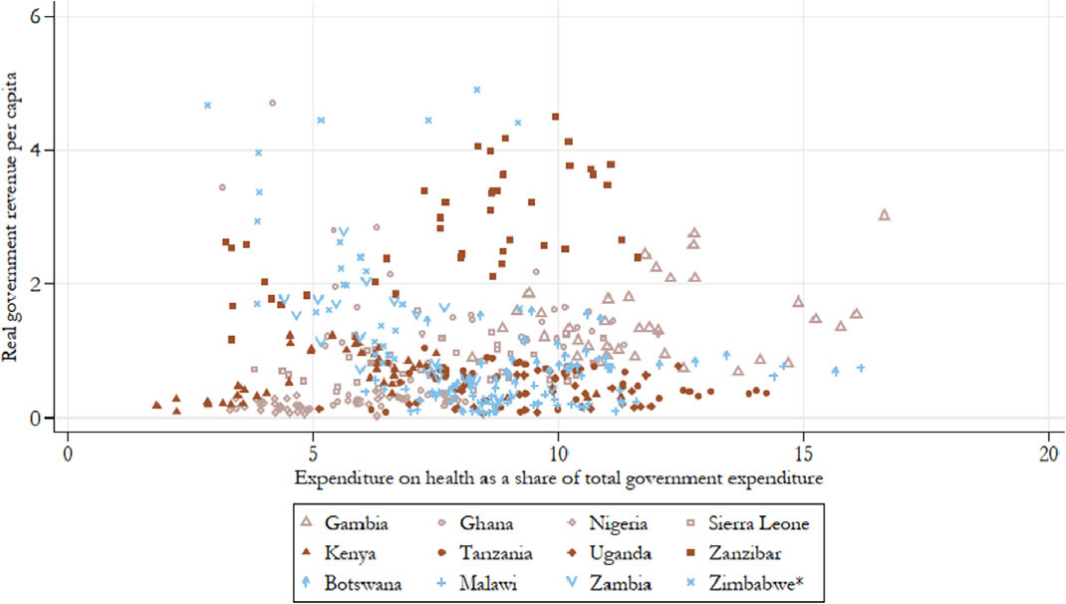
Real government revenue per capita and the share of the colonial budget dedicated to health spending, all years by country. *Note:* Authors’ calculations. Colour groups represent regions.

Since we anticipate colonial financial capacity to be one of, if not the most important factor shaping the expansion of health services in our sample countries, it is useful to consider how much revenue colonial governments had at their disposal and how much of total government spending was dedicated to health. As a share of total government expenditure, expenditure on health can be seen in Figure 2, plotted against real total government revenue per capita. Notably, the revenue estimates show a large variation in colonial budget size: with worst-performing Nigeria generating on average around £0.2 per annum per capita while the estimate for best-performing Zanzibar was close to £3 per annum per capita on average, a staggering 14-fold difference (see also Table 1). Excluding these two relative outliers, based on their respective size and population differences, East African colonies appear to have smaller budgets on average than West African colonies, in accordance with the findings of Frankema.^107^

**Table 1.** Dependent variable means

Period	GH	PC/GH (000’s)	IP	IP/POP (000’s)	OP	OP/POP (000’s)	GH	PC/GH (000’s)	IP	IP/POP (000’s)	OP	OP/POP (000’s)
*West Africa*												
			The Gambia						Ghana			
1900–19	2.000	109.311	597.273	3.365	8987.636	50.531	8.050	407.567	2832.550	0.926	32393.370	10.403
1920–39	3.800	58.576	1151.200	5.305	23921.100	108.819	39.600	103.706	15818.500	4.001	181619.100	46.342
1940–60	5.063	55.917	3340.750	11.568	100527.300	347.600	47.885	102.719	48184.500	9.303	615584.800	118.675
			Nigeria						Sierra Leone			
1900–19	11.025	2380.701	7673.075	0.389	61793.900	3.112	4.650	270.850	2850.200	2.498	20137.100	17.209
1920–39	31.750	767.370	38557.150	1.540	417030.300	16.578	4.950	300.345	4310.875	3.017	82452.230	57.624
1940–60	62.767	548.636	121534.300	3.704	1083213.000	33.051	6.000	278.468	11578.790	5.908	138617.500	71.492
*East Africa*												
			Kenya						Uganda			
1900–19	6.284	1106.737	17756.140	4.717	99872.710	26.506	16.000	213.928	2545.250	0.796	77764.440	24.271
1920–39	17.809	290.911	38358.450	8.681	264559.200	58.603	28.550	131.806	22161.080	5.772	644616.500	165.705
1940–60	45.312	123.862	144219.300	22.748	930962.300	145.743	38.250	130.811	74404.860	13.952	2348246.000	426.962
			Tanzania						Zanzibar			
1900–19	-	-	-	-	-	-	3.000	66.958	1275.500	6.017	12107.170	63.083
1920–39	38.118	159.201	30314.580	5.358	400658.700	69.742	3.350	86.744	3533.975	15.186	182730.000	763.903
1940–60	68.333	116.538	87979.100	10.758	1122565.000	139.821	5.789	51.254	6534.690	22.825	293751.300	1161.331
*Southern Africa*												
			Botswana						Malawi			
1900–19	-	-	-	-	-	-	5.563	510.089	-	-	-	-
1920–39	1.714	140.342	1106.000	3.590	41070.290	135.520	16.700	125.826	6324.789	2.905	253742.200	116.026
1940–60	5.143	90.519	7985.381	17.942	317009.900	722.414	21.095	137.993	27595.670	9.928	858190.100	308.520
			Zambia						Zimbabwe			
1900–19	-	-	-	-	-	-	-	-	-	-	792.286	0.559
1920–39	17.938	100.286	10205.440	5.617	182673.700	99.066	11.000	177.663	15503.530	7.793	43269.630	20.566
1940–60	26.143	91.661	30359.070	13.051	359149.100	163.471	15.263	181.651	83697.900	27.538	924778.900	312.251

*Note:* GH - mean government hospitals; PC/GH - mean population (000’s) to a government hospital

IP - mean government hospital inpatients; IP/POP (000’s) - mean government hospital inpatients per 1000 population OP - mean outpatient attendances; OP/POP (000’s) - mean outpatient attendances per 1000 population.

When we consider how countries prioritised spending on their medical departments relative to the size of their budgets it becomes evident that the capacity to spend did not necessarily determine greater spending on public goods. For example, neighbouring Tanzania and Kenya generated the same level of government revenue per capita (on average around £0.5 per annum per capita), yet Kenya devoted 5.6 per cent on average of its annual budget to healthcare spending, while Tanzania spent on average 9.4 per cent of its budget. The Gambia prioritised health spending more than any other colony, never devoting less than 8 per cent of total spending to health and reaching a peak of 16 per cent in 1940 and 1941. Furthermore, it had the budget to back it up, generating close to £1.5 per capita in annual revenue. In contrast, Malawi, Tanzania, Uganda, and Botswana also prioritised health spending to a greater extent (around 9 per cent on average) but their revenue-generating abilities were far more limited, bringing in between £0.2–0.6 per annum per capita on average. Irrespective of smaller budgets on average, East African colonies increased their budgets allocated to healthcare faster compared with colonies in West Africa.^108^ ^,^^109^

**Figure 3.**
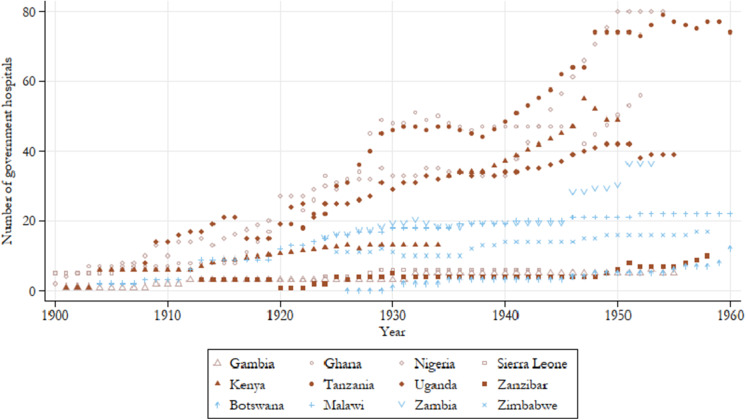
Annual count of government hospitals, all years by country. *Note:* Authors’ calculations. Colour groups represent regions.

**Figure 4.**
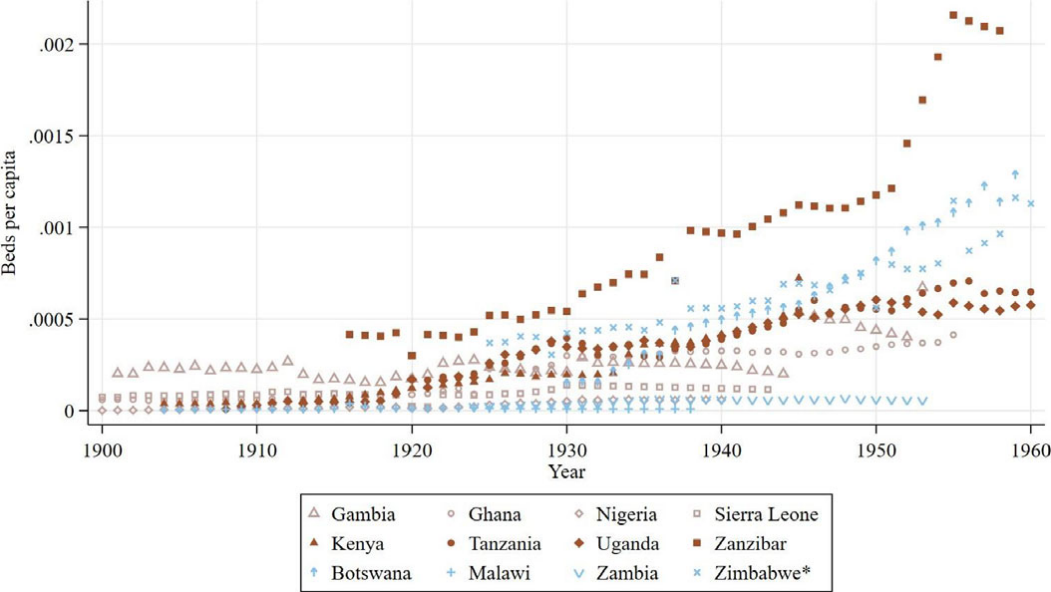
Annual count of beds for African patients at government hospitals per capita, all years by country. *Note:* Authors’ calculations. Colour groups represent regions.

To get a sense of the nominal expansion in government hospitals, Figure 3 shows the annual stock of government hospitals, not accounting for the growth in the domestic population over the period. Tanzania is one of the top performers, undergoing a rapid expansion in hospitals during the 1920s, which was followed by a period of stagnation in the 1930s and another surge in hospital building during the 1940s. Ghana’s initial expansion started a few years earlier (circa 1918) but mirrors this trajectory to a large extent, though its period of stagnation extends further into the 1940s with its second surge emerging in the later years of that decade. Nigeria shows the greatest response to the 1940 Colonial Development and Act, undergoing a substantial expansion in hospitals starting in that year, with the stock of hospitals more than doubling in the decade which followed. Uganda, Malawi, and Zambia followed a more modest yet steady expansion throughout the period while Botswana, The Gambia, Sierra Leone, Zanzibar, and Zimbabwe showed little development in this regard, and no clear regional patterns emerge. Figure 4 shows the annual count of beds reserved for African patients at government hospitals per capita. Here a more pronounced regional pattern emerges, where after 1920, all East African colonies provided more beds per capita compared with West African colonies although The Gambia comes close to the average East African trend. Meanwhile variation in the Southern region is much larger with Malawi and Zambia providing the least beds of all British colonies, and Zimbabwe and Botswana providing more beds per capita than all other West African colonies.

**Figure 5.**
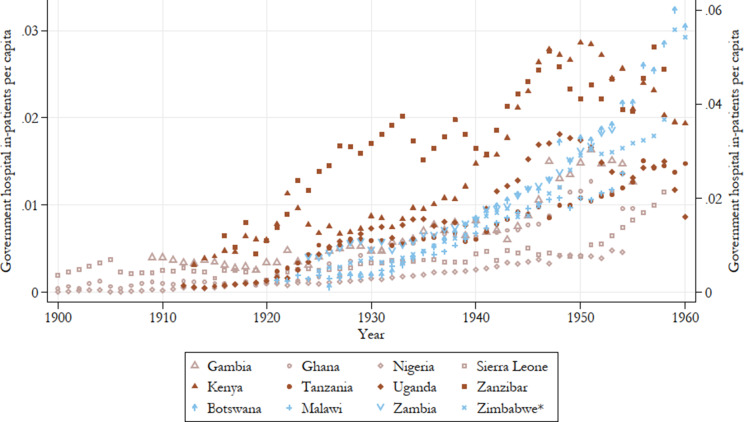
Government hospital inpatients per capita, all years by country. *Note:* Zimbabwe on the left axis. Other countries are on the right axis. Authors’ calculations. Colour groups represent regions.

**Figure 6.**
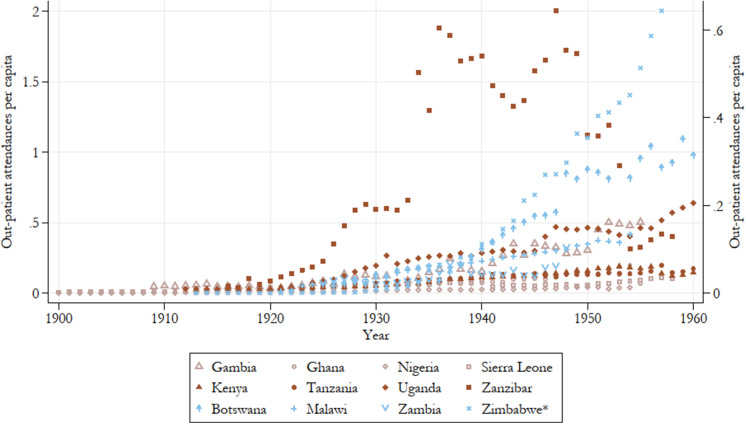
Outpatient attendances per capita, 1900–60. *Note:* Zimbabwe on the left axis. Other countries are on the right axis. Authors’ calculations. Colour groups represent regions.

In terms of the number of inpatients in government hospitals accounting for population growth (Figure 5) a much clearer regional pattern is visible: Except for Zimbabwe’s performance, which is somewhat inflated given the very high inpatient numbers it reports for the last two years of the 1950s, East African colonies are mostly reporting higher utilisation of their government hospitals. Southern African colonies as a group largely come in second, with Botswana and Zimbabwe only outperforming the East African group by the end of the period. West African colonies, despite having longer experience in healthcare provision, lag behind the rest in terms of patient turnover. Across the board, however, the number of government hospital inpatients is increasing over the period with a particularly sharp and consistent uptick in Southern African colonies beginning in the early 1930s. The picture of increasing utilisation is similar, though a bit less clear in the estimates of outpatient attendance over time (Figure 6). The uptake of these services is notably later than that of inpatients at government hospitals, indicative of the expansion of health services into the underserved rural areas following the second CDA.

**Figure 7.**
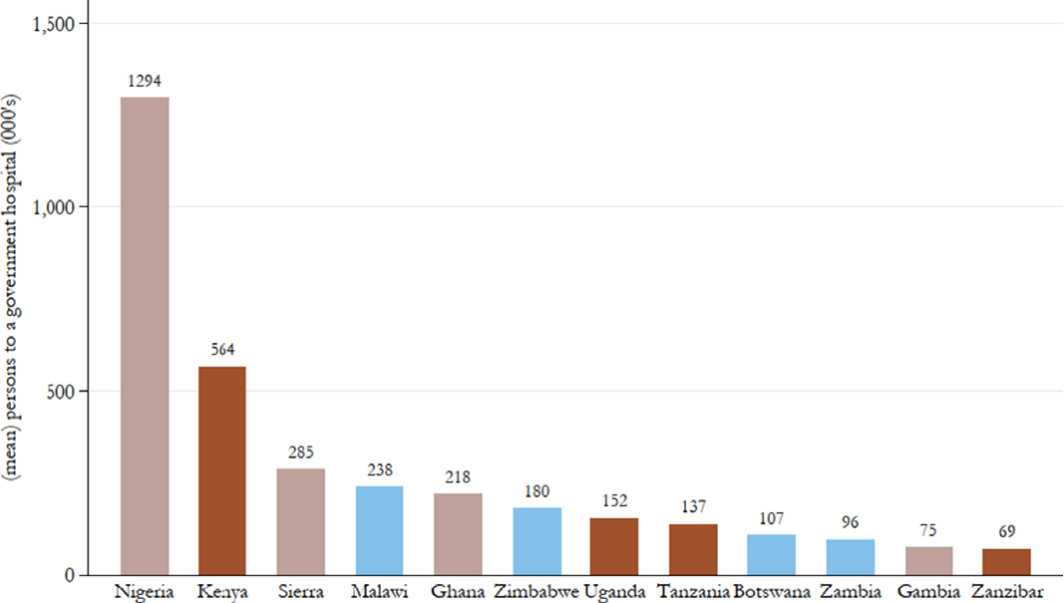
Mean persons (000’s) to a government hospital, 1900–60. *Note:* Authors’ calculations. Colour groups represent regions.

**Figure 8.**
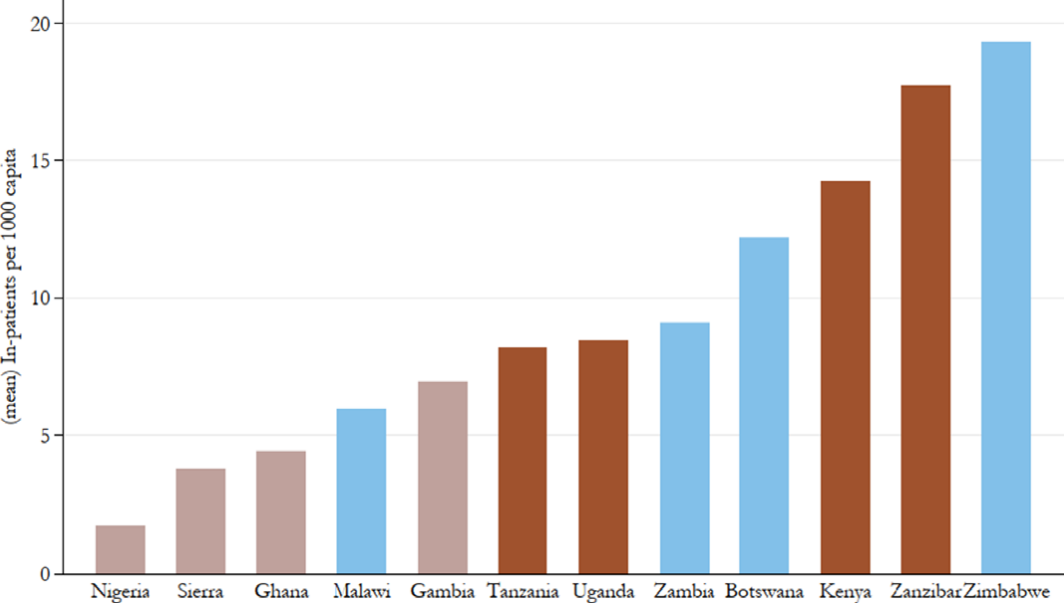
Mean government hospital inpatients per 1000 capita, 1900–60. *Note:* Authors’ calculations. Colour groups represent regions.

**Figure 9.**
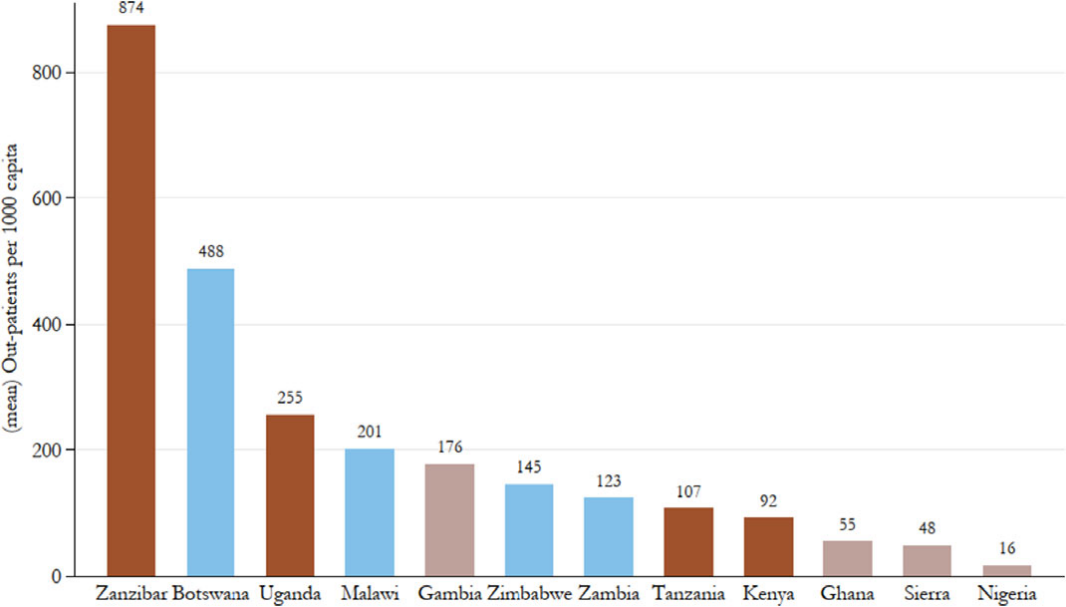
Mean outpatient attendances per 1000 capita, 1900–60. *Note:* Authors’ calculations. Colour groups represent regions.

Figures 7, 8, and 9 show means over the study period for government hospitals per capita, government hospital inpatients per capita, and outpatient attendances per capita, clearly showing large heterogeneity of public healthcare provision across British Africa. In terms of the scope of government hospital provision relative to the size of the population, it is not surprising that Nigeria, with its very large population, is the worst-performing colony with close to 1.3 million persons to a government hospital. This is double the next worst-performing colony, Kenya, where there were on average 564 000 persons in a government hospital. Except for The Gambia, the scope of West African colonies’ government hospital provision appears lower than East and Southern African colonies on average.

## Results: Healthcare system performance

Tables 2 and 3 present the estimates from our two main models aiming to test the association between various supply factors and the performance of public health systems over time. Table 2 contains our two sub-models of *scope*, with column 1 presenting the results for the model with log government hospitals beds reserved for African patients per capita as the dependent variable, and column 2 presenting the results for the model with log government hospitals per capita.

**Table 2.** Models of scope

	(1) Beds per capita	(2) Hospitals per capita
*Region*		
West African colony	Ref.	Ref.
East African colony	0.458***	1.137***
	(0.102)	(0.088)
Southern African colony	–0.832***	1.477***
	(0.152)	(0.142)
*European settler share*		
Non-settler colony	Ref.	Ref.
Settler colony	–0.186*	–0.561***
	(0.108)	(0.108)
*Alternative providers*		
Mission hospitals per capita	–0.145***	0.031
	(0.029)	(0.032)
*Period*		
1900–19	Ref.	Ref.
1920–39	–0.138	–0.425***
	(0.086)	(0.086)
1940–60	0.315**	–0.666***
	(0.135)	(0.137)
Real TGR per capita	0.366***	0.666***
	(0.070)	(0.066)
TME share of TGE	–0.020	0.090***
	(0.014)	(0.014)
Medical department est.	–0.187**	0.606***
	(0.076)	(0.072)
European staff per capita	0.503***	–0.009
	(0.049)	(0.059)
State hospitals per capita	0.822***	
	(0.055)	
Average no. beds per hospital		–0.194***
		(0.060)
Country area	0.000***	–0.000
	(0.000)	(0.000)
Constant	5.178***	–14.150***
	(1.135)	(0.902)
Observations	304	304
r2_w	0.469	0.267
r2_b	0.975	0.897

*Note:* State hospitals and hospital beds exclude those designated for Europeans.

All country average ratio method applied for exclusions.

All continuous variables are logged.

Standard errors in parentheses.

* *p*<0.10, ** *p*<0.05, *** *p*<0.01

**Table 3.** Models of utilization

	(1) Inpatients per capita	(2) Outpatients per capita
*Region*		
West African colony	Ref.	Ref.
East African colony	0.844***	1.602***
	(0.082)	(0.133)
Southern African colony	0.940***	1.987***
	(0.128)	(0.207)
*European settler share*		
Non-settler colony	Ref.	Ref.
Settler colony	0.243***	–0.938***
	(0.083)	(0.135)
*Alternative providers*		
Mission hospitals per capita	0.138***	0.138***
	(0.025)	(0.040)
*Period*		
1900–19	Ref.	Ref.
1920–39	0.394***	0.759***
	(0.068)	(0.110)
1940–60	0.454***	0.762***
	(0.107)	(0.173)
Real TGR per capita	0.406***	0.621***
	(0.058)	(0.094)
TME share of TGE	–0.007	0.108***
	(0.011)	(0.018)
Medical department est.	0.426***	0.289***
	(0.060)	(0.097)
European staff per capita	–0.137***	0.034
	(0.045)	(0.073)
State hospitals per capita	0.208***	–0.037
	(0.044)	(0.070)
Average no. beds per hospital	0.319***	0.237***
	(0.047)	(0.077)
Country area	–0.000	–0.000*
	(0.000)	(0.000)
Constant	–5.827***	–4.796***
	(0.920)	(1.485)
Observations	295	295
r2_w	0.746	0.725
r2_b	0.992	0.957

*Note:* State hospitals and state hospital beds exclude those designated for Europeans.

All country average ratio method applied for exclusions.

All continuous variables are logged.

Standard errors in parentheses.

* *p*<0.10, ** *p*<0.05, *** *p*<0.01

Table 3 contains our two sub-models of utilization with column 1 presenting the results for the model of log inpatients per capita as the dependent variable, and column 2 presenting the results for the model with log outpatients per capita as the dependent variable. We start by discussing the results pertaining to our hypothesis and end with the results for the other covariates: size of budgets, number of medical staff and year of medical department establishment.

### Regional diversity in healthcare provision

**Figure 10.**
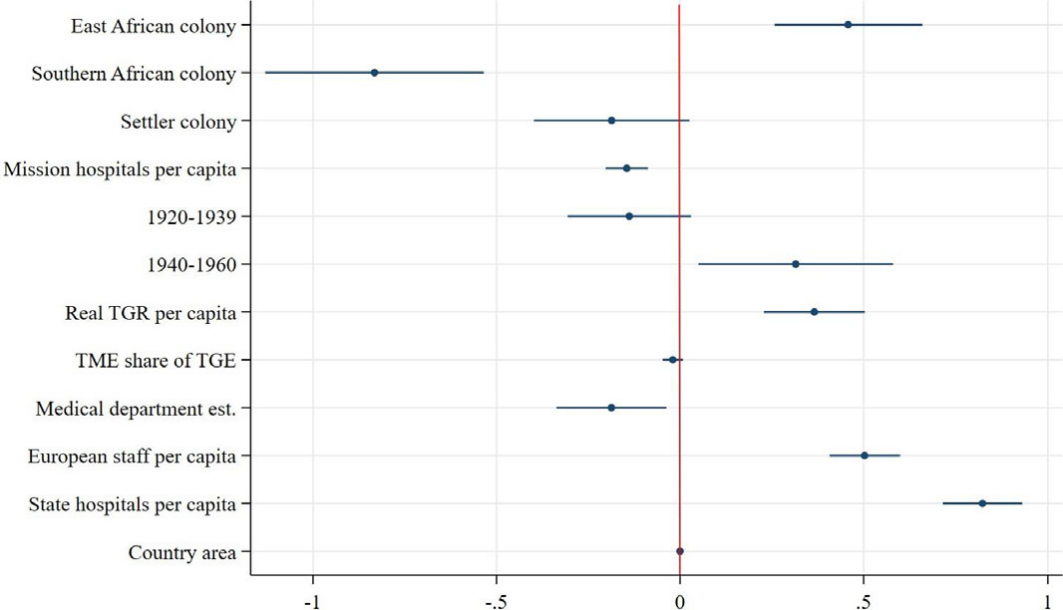
Coefficient plot of model with log government hospital beds per capita as dependent variable.

**Figure 11.**
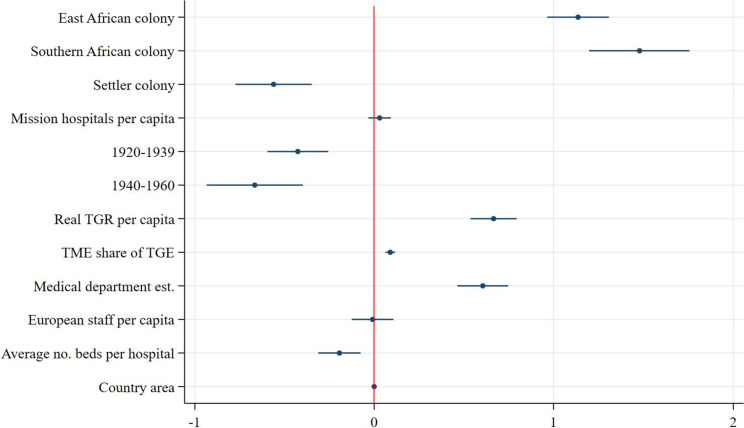
Coefficient plot of model with log government hospitals per capita as dependent variable.

**Figure 12.**
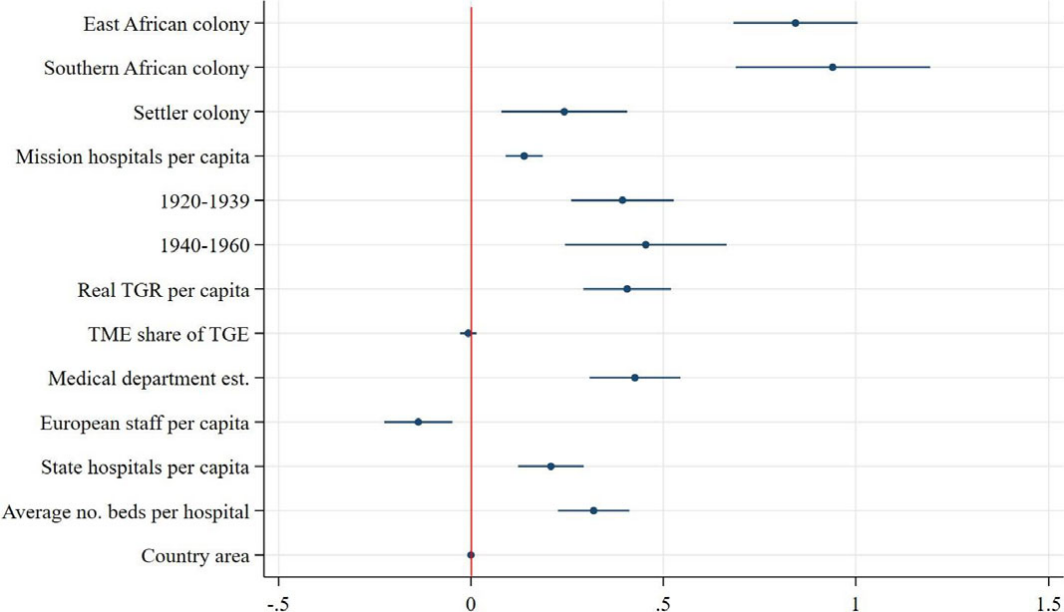
Coefficient plot of model with log government hospital inpatients per capita as dependent variable.

**Figure 13.**
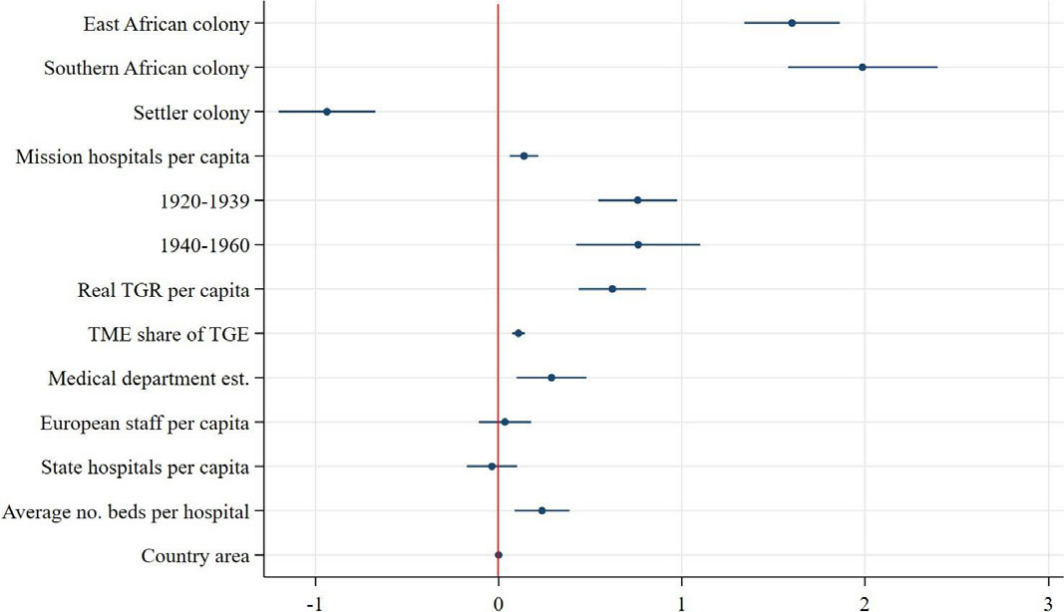
Coefficient plot of model with log outpatient attendances per capita as dependent variable.

Speaking to our first hypothesis, we find that East African colonies outperformed West African colonies in all measures of scope and utilisation. Southern African colonies outperformed West African colonies in all measures except for the number of beds for African patients at state hospitals (see Figure 10). From the coefficient plots of the remaining models (Figures 11–13), we can see that East African colonies and Southern African colonies performed similarly in terms of the number of government hospitals per capita and on both measures of utilisation (in- and outpatients). Since we control for differences in budget; the relative prioritisation of health spending in terms of total spending; the availability of medical staff; and years since first contact with Western medicine/experience with healthcare provision (proxied by the date of establishment of a colonial medical department per colony) these findings suggest that East and South African colonies were generally providing more health services (beds and hospitals per capita) than West African colonies despite their relative budget constraints and shorter experience in providing public healthcare.

Additionally, due to differences in budget, staff and exposure to Western medicine, West Africa was thought to have a harsher disease environments particularly for Europeans compared with East and Southern Africa.^110^ The fact that West African colonies built fewer hospitals and reached a smaller share of their population with state-sponsored healthcare could be due to the fact that relatively more resources and attention were given to improving European health throughout the colonial period. Furthermore, the regional dummy could be capturing regional differences in, for example, demand for healthcare, where general acceptance of and demand for healthcare rose faster in East and Southern Africa, perhaps partly due to the larger role of missionaries which were instrumental in swaying public opinion in favour of European-provided healthcare services.^111^

### Healthcare provision in settler versus non-settler colonies

In line with our second hypothesis, the models of scope show that colonial authorities in settler colonies supplied substantially fewer beds for African patients in fewer government hospitals than authorities in non-settler colonies: the average number of beds for African patients at government hospitals per capita was 17 per cent^112^ lower in settler colonies and the average number of government hospitals per capita was 43 per cent^113^ lower in settler colonies. Within these hospitals, however, there were on average 28 per cent^114^ more inpatients per capita admitted per year compared with non-settler colony government hospitals, suggesting greater inpatient turnover in settler colony government hospitals, despite an often more pronounced antagonistic stance towards improving welfare for Africans. This could be because of greater public hospital efficiency or a greater population density in the regions where settler colonies chose to build their hospitals relative to non-settler colonies which may have been more decentralized and dispersed. As most European settlers resided in bigger (administrative) centres, government hospitals were primarily built in those cities. Within settler colonies, that geographical pattern remained more persistent compared with non-settler colonies where during the colonial period there was a clearer shift towards healthcare provision in rural areas, prominently so in Tanganyika.^115^ This can also be seen from the result of the utilisation of outpatient services (Figure 13), which in settler colonies was 61 per cent lower on average than in non-settler colonies, lending credence to the notion that non-settler rural service provision was more geographically widespread.^116^

### Alternative suppliers and government healthcare provision

The models of scope (Figures 10–11) show that an increase in the number of missionary hospitals corresponds with a decrease in the number of beds provided in state hospitals, though the number of state hospitals is not statistically significantly correlated with an increase in the number of mission hospitals.

This supports the idea that missionary healthcare provision was an important substitute for state-supplied healthcare. Especially in East Africa, colonial administrations considered medical missions to be a necessary part of local colonial institutions.^117^ At the same time, colonial authorities were aware of the competition of medical missions. In 1921, the medical director of Kenya noted that ‘a government hospital is a tangible sign of government activities which is understood by every native but it is doubtful whether a subsidized mission hospital is in any way connected in the minds of the majority of the patients a being anything more than a token of the benevolence of the missionaries who therefore reap the credit and the resulting influence.’^118^ This made medical departments reluctant to continue subsidizing missions and invested instead in their own healthcare provisions.

In line with expectations, both models of utilisation show that an increase in the number of mission hospitals is associated with an increase in the number of inpatients and outpatients per capita treated in state hospitals.^119^

### Colonial governments’ healthcare provision and external (global) factors

Despite substantial between-colony variation, healthcare provision increased everywhere in colonial British Africa throughout the colonial period, in terms of the availability of hospital beds, the number of hospitals built as well as in the number of patients treated, see Figures 3–5. However, while the number of government hospitals increased in all colonies, this progress could not keep up with the growth of African populations. Our second model of scope (Figure 11) reveals a significant decrease in the expansion of government hospitals per capita over time. The growing awareness of the importance of improving the health of colonial populations coupled with a broader development agenda after the 1940s did not result in a greater availability of hospitals per capita but did result in a greater availability of hospital beds. These expansionary efforts also resulted in an increase in in- and outpatient services over time, as our models of utilisation suggest (Figures 12 and 13). The largest increase in both inpatients and outpatients treated is found for the period after 1940, which is likely driven by the increase in the supply of healthcare, but might also be a reflection of the increase in acceptance of and demand for Western medicine.^120^ Finally and not surprisingly, colonies with more and larger hospitals were able to treat more inpatients (Figure 12), but not outpatients (Figure 13) who were treated in smaller medical facilities such as dispensaries, and did not stay overnight.

### The size of the budgets and medical staff

As discussed before, the two most stringent factors constraining the colonial government’s capacity to provide health services were the size of the budget and the number of medical personnel. Our estimations show that indeed, colonial budgets are always positively and significantly associated with both scope and utilisation of government health services. Furthermore, the share of the budget devoted to health services, at least partly signalling priority given to health, is positively associated with the expansion of government hospitals per capita and outpatient attendance, but not statistically different from zero in terms of beds and inpatients. This is likely since spending on the medical department is directly linked to hospital infrastructure and personnel remuneration which is in turn linked to utilisation, both of which are already controlled for separately in this model. The number of European staff in the medical department is positively associated with the number of beds for African patients, confirming our expectations that European medical staff was a direct supply constraint to the size of hospitals. However, the negative association between European medical staff and inpatients in the model of utilisation implies that European staff was less of a constraint in the treatment of inpatients suggesting an increasing role for African medical staff, an element that our current data and model are unable to capture. Regional variation in the Africanization of the medical service, however, is an important area for future research.

Finally, in our model of scope (table 3) we control for the size of hospitals (average number of beds) and year of establishment of the medical department and in our models for utilisation we control for both the number of government hospitals (per capita), the size of hospitals and year of establishment of the medical department in each colony. The negative coefficient on average beds per hospital in the model of scope suggests that hospital quantity and size are inversely related. In the first model of utilization (inpatients, Figure 12), the number of beds per government hospital and the number of government hospitals are both positively associated with the number of inpatients treated in said hospitals. In the outpatient model (Figure 13), hospital size is positively associated with outpatient attendance, since a greater capacity to treat inpatients might be related to a greater capacity to treat outpatients. The number of hospitals per capita has a negative association with the number of outpatients treated, although the coefficient is not significant and very small. More government hospitals were able to provide at least some in– and outpatient care in addition to the outpatient care taking place at rural dispensaries. Finally, experience in providing healthcare (proxied for by the year of establishment of the medical department) is positively and significantly associated with more healthcare provision, both in terms of scope and in terms of utilisation.

## Robustness tests

In the main specification, we include both log real total government revenue per capita as a measure of a country’s overall budget and log share of total government expenditure that is devoted to the medical department as a measure of the administration’s prioritisation of public health relative to other fiscal expenditures. As total government revenue and total expenditure on the medical department could be correlated, we drop total government revenue and include only total medical expenditure. Appendix B and Tables B1 and B2 contain these specifications. The results mirror those in our main specification.

To account for differences in country size we re-estimate our model of scope using government hospitals per square kilometre instead of government hospitals per capita. Tables B3 and B4 contain the results. Here East Africa is still performing better than West Africa but Southern Africa is not.

To account for the potentially distorting effects of very small countries/islands, we re-estimate our main models with various exclusions: excluding both Zanzibar and The Gambia (Tables B5 and B6); excluding only Zanzibar (Tables B7 and B8); excluding only The Gambia (Tables B9 and B10). The results are largely similar to our main specification with the only model affected by the respective exclusions being the inpatients model, within which the region dummies and European staff variables lose significance. The significance of the region dummies is only lost if both Zanzibar and The Gambia are excluded.

## Conclusion

Despite facing continuous financial and personnel shortages throughout the colonial period, we find increasing healthcare provision in British colonial Africa, both in terms of scope and in terms of utilization, especially after the 1920s. This reflects the changing emphasis of colonial policies after the First World War towards broader state responsibility with regard to social and economic development which was formalised by the adoption of the Colonial Development Act in 1929.^121^ The largest expansion in healthcare provision, however, took place only after the Second World War, when Britain broadened its development agenda, and Western biomedicine gained wider acceptance in Africa. At the same time, we find substantial variation between countries and over time in terms of the number and geographical dispersion of healthcare facilities, the number of medical personnel employed, and the number of patients treated, both in absolute terms and as a share of the population.

Our evidence suggests a divide between East and West Africa in terms of medical budgets and access to healthcare facilities. Despite generally smaller government budgets, East African countries spent relatively more on healthcare compared with most West African countries. Furthermore, healthcare facilities in East Africa were on average smaller in size yet more geographically dispersed and provided basic medical services to a larger share of the population compared with the more centralised healthcare provision in West Africa. This could be the result of a more hostile disease environment, requiring more resources to be spent on European healthcare, or it might reflect differences in for example demand between regions.

While initially most healthcare was provided for the European settler population in all colonies, a larger settler population and the associated larger investments in European healthcare in settler colonies only led to a partial trickledown effect. While in settler colonies fewer hospitals were built to serve the general population, the utilization of hospital care (inpatients per capita) was substantially higher compared with non-settler colonies. As most European settlers resided in larger (administrative) centres, government hospitals were primarily built in those cities all over colonial Africa and the shift towards rural healthcare was much smaller in settler colonies. The finding that in settler colonies, fewer outpatients received treatment, confirms the focus of healthcare in urban centres compared with rural outposts.

The role of missionaries as alternative providers of healthcare is associated with the building of more hospitals, which supports the idea that missionary healthcare provision was an important catalyst for the expansion of the public health sector. However, colonies with high missionary presence treated fewer patients, both in government hospitals and at rural dispensaries, although this effect is non-significant. This likely reflects the fact that missionaries were successful in encouraging acceptance of Western healthcare by fostering close contact with local societies. It also suggests that alternative healthcare providers were able to ease some of the healthcare burden in terms of the treatment of patients that colonial governments were facing.

## Supporting information

Bolt and Cilliers supplementary material Bolt and Cilliers supplementary material

## Footnotes

1 Stephen Addae, *The evolution of modern medicine in a developing country: Ghana 1880–1960* (Bishop Auckland, Durham Academic Press, 1997); Jean Germain Gros , Healthcare Policy in Africa: Institutions and Politics from Colonialism to the Present (Lanham, MD: Rowman & Littlefield, 2015), 41.10.1080/13623699.2016.114241426882872

2

Angus Deaton , The Great Escape: Health, Wealth, and the Origins of Inequality (Princeton, NJ: Princeton University Press, 2013); Tamara Giles-Vernick et al., ‘Social History, Biology, and the Emergence of HIV in Colonial Africa’, The Journal of African History, 54, 1 (2013), 11–30 ; James L. A. Webb , ‘Historical Epidemiology and Infectious Disease Processes in Africa’, The Journal of African History, 54, 1 (2013), 3–10 .

3

Rebekah Lee , Health, Healing and Illness in African History (London: Bloomsbury Academic Publishing, 2021), 81.

4

Daron Acemoglu , Simon Johnson , and James A Robinson , ‘The Colonial Origins of Comparative Development: An Empirical Investigation’, American Economic Review, 91, 5 (2001), 1370 ; Crawford Young , The African Colonial State in Comparative Perspective (New Haven, CT: Yale University Press, 1994).

5

Jeffrey Herbst , States and Power in Africa: Comparative Lessons in Authority and Control (Princeton, NJ: Princeton University Press, 2000).

6

John Iliffe , East African Doctors, A History of the Modern Profession (Cambridge: Cambridge University Press, 1998); Lee, *op. cit.* (note 3) 78, argues that Indigenous healthcare regimes remained prevalent, offering local populations access to health services throughout the colonial period, although over time, the basis of the power of traditional healers was substantially eroded.

7 Ewout Frankema, ‘Raising Revenue in the British Empire, 1870–1940: How ‘Extractive’ Were Colonial Taxes?’, *Journal of Global History*, 5, 3 (2010), 142, Table 3.

8

Bill Warren , Imperialism: Pioneer of Capitalism (Brooklyn, NY: Verso Books, 1980). During the first decades of colonial rule, the health situation on the continent deteriorated, particularly in Eastern Africa, Iliffe, *op. cit.* (note 6), 28; Lee, *op. cit.* (note 3) 68.

9 K. David Patterson, *Health in Colonial Ghana: Disease, Medicine, and Socio-economic Change, 1900-1955.* (New York: Crossroads Press, 1981), 11; Addae, *op. cit.* (note 1), 40, 55; Gros, *op. cit.* (note 1), 46.

10 Largely aimed at improving the health of Europeans until 1920, see context section.

11 Florence Ejogha Nkwam, ‘British Medical and Health Policies in West Africa c.1920-1960.’ (unpublished PhD thesis: University of London School of Oriental and African Studies, 1988); Gros, *op. cit.* (note 1), 47. Lee, *op. cit.* (note 3), 77.

12 The territories studied are Bechuanaland (Botswana), Gold Coast (Ghana), The Gambia, East African Protectorate (Kenya), Nigeria, Northern Rhodesia (Zambia), Nyasaland (Malawi), Sierra Leone, Southern Rhodesia (Zimbabwe), Tanganyika (Tanzania), Uganda, and Zanzibar.

13 Lee, *op. cit.* (note 3), 26.

14 Iliffe, *op. cit.* (note 6), 10.

15 Lee, *op. cit.* (note 3), 29.

16

Gerald W. Hartwig and K. David Patterson , Disease in African History: An Introductory Survey and Case Studies (Durham, NC: Duke University Press, 1978), 5–7 ; Rita Headrick and Daniel R. Headrick , Colonialism, Health and Illness in French Equatorial Africa, 1885-1935 (Atlanta, GA: African Studies Association Press, 1994), 29 .

17 Lee, *op. cit.* (note 3), 68.

18

Steven Feierman and John M. Janzen , The Social Basis of Health & Healing in Africa (Berkeley, CA: University of California Press, 1992); Meredeth Turshen , ‘The Impact of Colonialism on Health and Health Services in Tanzania,’ International Journal of Health Services: Planning, Administration, Evaluation, 7, 1 (1977), 7–35 319069 10.2190/L9G4-KJVK-AW7A-Q9JD.

19 Hartwig and Patterson, *op. cit.* (note 16); Dennis D. Cordell and Joel W. Gregory, *African Population and Capitalism: Historical Perspectives* (Madison, WI: University of Wisconsin Press, 1994); Headrick and Headrick, *op. cit.* (note 16); Jan Vansina, *Being Colonized: The Kuba Experience in Rural Congo, 1880–1960* (Madison, WI: University of Wisconsin Press, 2010); Giles-Vernick et al., *op. cit.* (note 2); Webb, *op. cit.* (note 2).

20
*Ibid.*, 10; Iliffe, *op. cit.* (note 6), 28; Lee, *op. cit.* (note 3), 68.

21 Lee, *op. cit.* (note 3), 30; Iliffe, *op. cit.* (note 6), 11.

22 Addae, *op. cit.* (note 1). This is not to say that African healing practices did not play a role in shaping the development of colonial and post-independence public healthcare provision, but there was an important organizational difference between the two forms of healthcare provision: European-founded hospitals, clinics and dispensaries provided care for the sick which had no equivalent in the traditional healing systems. This colonial healthcare system also provided the fundament for post-independence public healthcare systems, see Mario J. Azevedo , The Colonial Medical System(s) and the Health of Africans. In: Historical Perspectives on the State of Health and Health Systems in Africa, Volume I. (Cham, Switzerland: Palgrave Macmillan, 2017).

23

Anna Greenwood , Beyond the State: The Colonial Medical Service in British Africa (Manchester, UK: Manchester University Press, 2015), 1.

24

Tamara Giles-Vernick, L.A. James and J.R. Webb , Global Health in Africa, Historical Perspectives on Disease Control (Athens, OH: Ohio University Press, 2013), 4.

25 Nkwam, *op. cit.* (note 11). Patterson, *op. cit.* (note 9).

26

Colin Baker , ‘The Government Medical Service in Malawi: An Administrative History, 1891–1974,’ Medical History, 20, 3 (1976), 296 792586 10.1017/s0025727300022687PMC1081783; Michael Gelfand, *A Service to the Sick: A History of the Health Services for Africans in Southern Rhodesia (1890–1953)* (Gwelo: Mambo Press, 1976); Michael Chanda Chiseni, ‘Here I Am Send Me: The Historical and Long-term Impact of Christian Missionaries in Zambia 1924-2018’ (unpublished PhD thesis: Lund University, 2022), 165; M. H. Webster , ‘The Medical Services of Rhodesia,’ Canadian Journal of Public Health, 57, 3 (1966), 117 5907575 .

27

William Malcolm Hailey , An African Survey: A Study of Problems Arising in Africa, South of the Sahara (Oxford: Oxford University Press, 1938), 1122 .

28 Nkwam, *op. cit.* (note 11), 18; Patterson, *op. cit.* (note 9).

29 Baker, *op. cit.* (note 26).

30 Turshen, *op. cit.* (note 18); Michael Jennings , ‘Healing of Bodies, Salvation of Souls’: Missionary Medicine in Colonial Tanganyika, 1870s-1939,’ Journal of Religion in Africa, 38, 1 (2008), 27–56 ; Ann Beck, ‘Medicine and Society in Tanganyika, 1890-1930; A Historical Inquiry.’ Transactions of the American Philosophical Society, 67, 3 (1977), 1–59 .

31

K. David Patterson , Infectious Diseases in Twentieth-century Africa: A Bibliography of Their Distribution and Consequences (Waltham, MA: Crossroads Press, 1979); Patterson, *op. cit.* (note 9).

32 Gelfand, *op. cit.* (note 26).

33

W. D. Foster , ‘Robert Moffat and the Beginnings of the Government Medical Service in Uganda,’ Medical History, 13, 3 (1969), 237–504893623 10.1017/s0025727300014502PMC1033951.

34

Heather Bell , Frontiers of Medicine in the Anglo-Agyptian Sudan, 1899-1940 (Oxford: Oxford University Press, 1999).

35 T.S. Gale, ‘Official Medical Policy in British West Africa, 1870–1930’ (PhD thesis, University of London School of Oriental and African Studies, 1970); Beck, *op. cit.* (note 30); Ryan Johnson , ‘The West African Medical Staff and the Administration of Imperial Tropical Medicine, 1902–14,’ The Journal of Imperial and Commonwealth History, 38, 3 (2010), 419–3920722228 10.1080/03086534.2010.503396.

36 Beck, *op. cit.* (note 30).

37 Nkwam, *op. cit.* (note 11).

38 Gale, *op. cit.* (note 35); Johnson, *op. cit.* (note 35).

39 Anna Crozier, *Practising Colonial Medicine: The Colonial Medical Service in British East Africa.* (London: I.B. Tauris, 2014).

40 Anna Greenwood, *op. cit.* (note 23).

41 Lee, *op. cit.* (note 3).

42 Giles-Vernick and Webb, *op. cit.* (note 24).

43 Philip D. Curtin, ‘Medical Knowledge and Urban Planning in Colonial Tropical Africa’, in Feierman and Janzen, *op. cit.* (note 18).

44

George C. Abbott , ‘A Re-Examination of the 1929 Colonial Development Act,’ The Economic History Review, 24, 1 (1971), 68–81 ; Leigh A. Gardner , ‘Decentralization and Corruption in Historical Perspective: Evidence from Tax Collection in British Colonial Africa,’ Economic History of Developing Regions, 25, 2 (2010), 213–36.

45 The sleeping sickness epidemic in Uganda likely started in the late 1890s and peaked in 1902 and the outbreak lasted until 1920, Lea Berrang-Ford, Martin Odiit, Faustin Maiso, David Waltner-Toews, John McDermott J. ‘Sleeping sickness in Uganda: Revisiting Current and Historical Distributions’, *African Health Science*, (2006) 223–31.

46 Beck, *op. cit.* (note 30), 71.

47 Nkwam, *op. cit.* (note 11); Patterson, *op. cit.* (note 9); Beck, *op. cit.* (note 30); Gelfand, *op. cit.* (note 26); Bell, *op. cit.* (note 34).

48 The colonial Development Public Health Committee (CDPHC) observed that ‘[i]ndifferent health, whether from disease or malnutrition reduces the capacity for work. This depresses the standard of living of the inhabitants and prevents the proper development of the resources of the Territory and the expansion of Government revenue on which depends improvement in the standard of administration and measures for improving the material and moral well-being of the people’, Report of the CDPHC, quoted in (Nkwam, *op. cit.* (note 11), 37.

49 Abbott, *op. cit.* (note 44); Gardner, *op. cit.* (note 44).

50 Addae, *op. cit.* (note 1); Eric Schneider, Soren Edvinsson, and Kota Ogasawara, ‘Did Smallpox Cause Stillbirths? Maternal Smallpox Infection, Vaccination and Stillbirths in Sweden, 1780-1839’, *Population Studies*, (2023), 1–16.

51 Patterson, *op. cit.* (note 9), 11.

52 Headrick and Headrick, *op. cit.* (note 16); Johnson, *op. cit.* (note 35); Nkwam, *op. cit.* (note 11); Beck, *op. cit.* (note 30); Baker, *op. cit.* (note 26).

53 Patterson, *op. cit.* (note 9); Gelfand, *op. cit.* (note 26).

54 Feierman and Janzen, *op. cit.* (note 18), 16; Lee, *op. cit.* (note 3), 77.

55 Nkwam, *op. cit.* (note 11); 129; Patterson, *op. cit.* (note 9), 23–4.

56
*Ibid.*, 41.

57
*Ibid.*, 46–7.

58 Beck, *op. cit.* (note 30).

59

Randall M. Packard , A History of Global Health: Interventions into the Lives of Other Peoples (Baltimore: Johns Hopkins University Press, 2016), 62.

60 Michael Marmot, *The Health Gap: The Challenge of an Unequal World* (London: Bloomsbury, 2015).

61 Gros, *op. cit.* (note 1).

62 Frederick Cooper, *Africa since 1940: The Past of the Present*, New Approaches to African History (Cambridge: Cambridge University Press, 2002), 31; Gros, *op. cit.* (note 1), 76. Nkwam, *op. cit.* (note 11).

63 Nkwam, *op. cit.* (note 11), 212.

64

Deon Filmer and Lant Pritchett , ‘The Impact of Public Spending on Health: Does Money Matter?,’ Social Science & Medicine 49, 10 (1999), 1309–2310509822 10.1016/s0277-9536(99)00150-1; Sanjeev Gupta , Marijn Verhoeven , and Erwin R Tiongson , ‘The Effectiveness of Government Spending on Education and Health Care in Developing and Transition Economies,’ European Journal of Political Economy, 18, 4 (2002), 717–37; Farasat A. S. Bokhari , Yunwei Gai , and Pablo Gottret , ‘Government Health Expenditures and Health Outcomes,’ Health Economics, 16, 3 (2007), 257–7317001737 10.1002/hec.1157; Eric Arthur and Hassan E. Oaikhenan , ‘The Effects of Health Expenditure on Health Outcomes in Sub-Saharan Africa (SSA),’ African Development Review, 29, 3 (2017), 524–36; Jaison Chireshe and Matthew K. Ocran , ‘Health care expenditure and health outcomes in sub-Saharan African countries,’ African Development Review, 32, 3 (2020), 349–61.

65 Both the number of state hospital beds and the number of state hospitals have been adjusted to exclude beds/hospitals designated for European only use. A more detailed account of these adjustments is given in Appendix A. In the data section we provide more details on what constitutes a hospital.

66 Johnson, *op. cit. (note 35);* ‘Nkwam, *op. cit.* (note 11); Baker, *op. cit.* (note 26).

67

Leigh A. Gardner , Taxing Colonial Africa: The Political Economy of British Imperialism, (Oxford: Oxford University Press, 2012), 24–5. Although this self-sufficiency policy officially ended with the implementation of the Colonial Development and Welfare Act of 1940, financial means for healthcare remained limited throughout the period.

68 Annual Medical Report, Nyasaland, 1928, 6.

69 Patterson, *op. cit.* (note 9), 13.

70
*Ibid.*; Beck, *op. cit.* (note 30).

71 Greenwood, *op. cit.* (note 23), 8.

72 Patterson, *op. cit.* (note 9), 12; Beck, *op. cit.* (note 30), 58; Iliffe, *op. cit.* (note 6), 28

73 Beck, *op. cit.* (note 30), 58

74 Patterson, *op. cit.* (note 9),16. Frankema, *op. cit.* (note 7).

75 Beck, *op. cit.* (note 30), 82; Iliffe, *op. cit.* (note 6), 42, 60.

76 The reporting on African medical workers in colonial employment is also rather crude, which hinders studying the Africanization of the medical service. An important area for future research is mapping the full range of African contributions to the medical service under colonial rule.

77

Ewout Frankema , ‘Colonial Taxation and Government Spending in British Africa, 1880-1940: Maximizing Revenue or Minimizing Effort?’, Explorations in Economic History, 48, 1 (2010), 136–49.

78 Hartwig and Patterson, *op. cit.* (note 16).

79 It brought together the medical departments of The Gambia, Sierra Leone, the Gold Coast, Lagos, Southern Nigeria and Northern Nigeria.

80

Philip D. Curtin , ‘‘The White Man’s Grave:’ Image and Reality, 1780-1850’, Journal of British Studies 1, 1 (1961), 94–110 .

81 Greenwood, *op. cit.* (note 23).

82 Beck, *op. cit.* (note 30), 13.

83 Greenwood, *op. cit.* (note 23), 6.

84 Hartwig and Patterson, *op. cit.* (note 16), 16–17; Beck, *op. cit.* (note 30), 10; Nkwam, *op. cit.* (note 11), 19; Megan Vaughan, *Curing Their Ills: Colonial Power and African Illness* (Cambridge: Polity Press, 1991).

85 Lee, *op. cit.* (note 3), 80.

86

Kenneth Good , ‘Settler Colonialism: Economic Development and Class Formation.’ The Journal of Modern African Studies 14, 4 (1976), 597–620 ; Jack Paine , ‘Democratic Contradictions in European Settler Colonies.’ World Politics, 71, 3 (2019), 542–85.

87 Iliffe, *op. cit.* (note 6); Lee, *op. cit.* (note 3).

88 Beck, *op. cit.* (note 30), 21; Gelfand, *op. cit.* (note 26); Nkwam, *op. cit.* (note 11).

89 Vaughan, *op. cit.* (note 84).

90 Beck, *op. cit.* (note 30); Gelfand, *op. cit.* (note 26); Nkwam, *op. cit.* (note 11).

91 Greenwood, *op. cit.* (note 23) 1.

92
*Ibid.*, 74. Lee, *op. cit.* (note 3), 65.

93 Frankema, *op. cit.* (note 7), 137.

94 Bechuanaland Annual Colonial Report (1928), 18.

95 Bechuanaland Annual Colonial Report (1929), 24–5.

96 Lee, *op. cit.* (note 3), 72–7 describes how African medical auxiliaries served as crucial intermediaries between local patient communities and colonial health institutions. Additionally, she argues that local perceptions and adaptations of colonial medical interventions influenced which medical therapies or technologies African patients chose to adopt, leading to significant regional variations in the uptake of colonial healthcare services.

97 Lee, *op. cit.* (note 3).

98 Léa Rouanet, ‘Three Essays About Health Progress and Economic Development in Africa’ (unpublished PhD thesis: École des Hautes Études en Sciences Sociales [EHESS]), 22.

99

James Duminy , ‘A Piecemeal Avalanche: the Uneven Topography of Statistics in Colonial Kenya, c. 1900 to 1952’, Urban Forum, 28, 4 (2017); Tom Westland , ‘How Accurate are the Prices in the British Colonial Blue Books?’ Economic History of Developing Regions, 37, 1 (2022), 75–99 10.1080/20780389.2021.1940946PMC882761735154817.

100 Feierman and Janzen, *op. cit.* (note 18), 9.

101 While in terms of patients admitted recording errors are very likely, we have no information on whether the error is systematically biasing the recordings in a certain direction. In case of random error in the recordings, valid conclusions can still be drawn.

102 In- and outpatient data refer to all races, however, given the small share of Europeans in the total population, we do not expect the introduction of significant bias to the utilisation results.

103 Price indices were adapted from Frankema and van Waijenburg’s barebones consumption basket (one adult male’s annual basket excluding rent, fuel, and light) adjusted upward to 2100 calories, with maize as the staple crop. Ewout Frankema and Marlous Van Waijenburg , ‘Structural Impediments to African Growth? New Evidence from Real Wages in British Africa, 1880–1965’, The Journal of Economic History, 72, 4 (2012), 895–926 . For Botswana, Bolt and Hillbom’s prices were used. Jutta Bolt and Ellen Hillbom , ‘Long-term Trends in Economic Inequality: Lessons from Colonial Botswana, 1921–74’, The Economic History Review, 69, 4 (2016), 1255–84. For Uganda, De Haas’s prices were used with millet as the staple crop. Michiel de Haas, ‘Measuring Rural Welfare in Colonial Africa: Did Uganda’s Smallholders Thrive?’ *The Economic History Review*, 70, 2 (2017), 605–31. For Tanzania, Klocke’s prices were used with mixed meal as the staple crop. Sascha Klocke, ‘Land, Labour, Legacies: Long-term Trends in Inequality and Living Standards in Tanzania’ (unpublished PhD thesis: Lund University, 2021). For Zambia, new baskets were generated following the Frankema and van Waijenburg method using prices from the Northern Rhodesia Blue Books and maize as a staple crop.

104 The index base year was set to 1926. Values for missing years were interpolated using the average annual growth rate. Feinstein’s consumer goods and services index supplemented the following missing years (based on being a close match to the preceding year’s trend), for The Gambia 1906–18, Kenya 1916–25, and Nigeria 1946–65. Zanzibar values were deflated using Tanzania’s index. Zimbabwe values were deflated using Zambia’s index. Basket indices were smoothed using five-year moving averages. African population estimates come from Frankema and Jerven. Ewout Frankema and Morten Jerven , ‘Writing History Backwards or Sideways: Towards a Consensus on African population, 1850–2010’, The Economic History Review, 67, 4 (2014), 907–31. Zanzibar population estimates come from the Annual Medical Reports (1916) and available censuses (1938, 1948, 1958).

105

Ewout Frankema , Erik Green , and Ellen Hillbom , ‘Endogenous Processes of Colonial Settlement. The Success and Failure of European Settler Farming in Sub-Saharan Africa’, Revista de Historia Economica / Journal of Iberian and Latin American Economic History, 34, 2 (2016), 237–65.

106 Following this definition, we classify Zimbabwe, Zambia, and Kenya as settler colonies.

107 Frankema, *op. cit.* (note 7).

108 Frankema, *op. cit.* (note 7), 145.

109 This could be indicative of a stronger desire on the part of colonial officials in East Africa to provide broader access to healthcare, or of greater employment and training of African staff. Exploring this is beyond the scope of the current paper.

110 Curtin *op. cit.* (note 80), 95.

111 Beck, *op. cit.* (note 30), 21; Gelfand, *op. cit.* (note 26); Nkwam, *op. cit.* (note 11); Vaughan, *op. cit.* (note 84).

112 (exp(–0.186) – 1) * 100%. See Table 2 for coefficients.

113 (exp(–0.561) – 1) * 100%. See Table 2 for coefficients.

114 (exp(0.243) – 1) * 100%. See Table 3 for coefficients.

115 Beck *op. cit.* (note 30).

116 (exp(–0.938) – 1) * 100%. See Table 3 for coefficients.

117 Beck, *op. cit.* (note 30), 19.

118 Annual Medical Report of the Colony and Protectorate of Kenya, 1921, 23.

119 Beck, *op. cit.* (note 30), 82.

120 Patterson, *op. cit.* (note 9). Gros, *op. cit.* (note 1), 76. Nkwam, *op. cit.* (note 11).

121 Abbott, *op. cit.* (note 44).

## Supplementary material

The supplementary material for this article can be found at http://doi.org/10.1017/mdh.2024.45.

## Appendix A

To account for the fact that European patients and African patients had access to different spaces and different levels of care we exclude hospitals which only provided care to European patients in our tally of government hospitals per capita and we exclude beds in hospitals which catered to both European and non-European patients that were reserved for Europeans only in our tally of government hospital beds per capita. We also exclude the population of Europeans in our denominator for per capita calculations.

To inform our respective exclusions we use the Blue Books, chapter ‘Hospitals’, ‘Question 1’ where the hospital name is taken to flag European-only hospitals, and from ‘Question 2’ we use ward information to flag European-only wards. This information is provided systematically for Kenya, Nigeria, Tanzania, Sierra Leone, Uganda, and Zanzibar.

For Ghana, we estimate the ratio of European to African hospitals and hospital beds for a few benchmark years only. A linear growth rate is applied to interpolate missing values, and the tallies are adjusted accordingly.

For Botswana, the ratio of European to African hospitals and hospital beds is taken from the Colonial Annual Reports (where available), and tallies are adjusted accordingly. For The Gambia, Malawi, Zambia, and Zimbabwe, neither hospital names nor ward information were detailed enough to inform such exclusions. In these cases, we adjust our tallies by the ratio of European to African hospitals and hospital beds over time, using i) the ratio of the nearest neighbour, and ii) the average ratio of all countries where the ratio could be calculated. Our models of scope and utilization show little sensitivity to the type of ratio applied. We therefore use the all-country average method as our main estimates (Tables 2 and 3), and the nearest neighbour adjusted series as Tables A1 and A2 below.

**Table A1.** Alternate models of scope

	(1) Beds per capita	(2) Hospitals per capita
*Region*		
West African colony	Ref.	Ref.
East African colony	0.436***	1.128***
	(0.102)	(0.088)
Southern African colony	–0.874***	1.484***
	(0.151)	(0.143)
*European settler share*		
Non-settler colony	Ref.	Ref.
Settler colony	–0.178*	–0.554***
	(0.108)	(0.109)
*Alternative providers*		
Mission hospitals per capita	–0.143***	0.034
	(0.029)	(0.032)
*Period*		
1900–19	Ref.	Ref.
1920–39	–0.152*	–0.426***
	(0.086)	(0.087)
1940–60	0.299**	–0.671***
	(0.135)	(0.137)
Real TGR per capita	0.356***	0.661***
	(0.070)	(0.066)
TME share of TGE	–0.017	0.091***
	(0.014)	(0.014)
Medical department est.	–0.196**	0.608***
	(0.076)	(0.072)
European staff per capita	0.509***	–0.014
	(0.049)	(0.060)
State hospitals per capita	0.836***	
	(0.055)	
Country area	0.000***	–0.000
	(0.000)	(0.000)
Average no. beds per hospital		–0.179***
		(0.060)
Constant	5.483***	–14.226***
	(1.132)	(0.907)
Observations	304	304
r2 w	0.464	0.265
r2 b	0.976	0.898

*Note:* State hospitals and hospital beds for patients of all races.

Nearest neighbour ratio applied for country adjustments.

All continuous variables are logged.

Standard errors in parentheses.

* *p*<0.10, ** *p*<0.05, *** *p*<0.01

**Table A2.** Alternate models of utilization

	(1) Inpatients per capita	(2) Outpatients per capita
*Region*		
West African colony	Ref.	Ref.
East African colony	0.853***	1.613***
	(0.082)	(0.133)
Southern African colony	0.953***	1.992***
	(0.130)	(0.209)
*European settler share*		
Non-settler colony	Ref.	Ref.
Settler colony	0.240***	–0.942***
	(0.084)	(0.135)
*Alternative providers*		
Mission hospitals per capita	0.138***	0.137***
	(0.025)	(0.040)
*Period*		
1900–19	0.000	0.000
	(.)	(.)
1920–39	0.397***	0.760***
	(0.068)	(0.110)
1940–60	0.460***	0.767***
	(0.107)	(0.173)
Real TGR per capita	0.410***	0.627***
	(0.058)	(0.094)
TME share of TGE	–0.008	0.108***
	(0.011)	(0.018)
Medical department est.	0.429***	0.290***
	(0.060)	(0.098)
European staff per capita	–0.138***	0.037
	(0.046)	(0.073)
State hospitals per capita	0.202***	
	(0.044)	
Average no. beds per hospital	0.316***	0.228***
	(0.048)	(0.077)
Country area	–0.000	–0.000*
	(0.000)	(0.000)
State hospitals per capita		–0.043
		(0.070)
Constant	–5.921***	–4.848***
	(0.927)	(1.496)
Observations	295	295
r2 w	0.745	0.724
r2 b	0.992	0.957

*Note:* State hospitals and state hospital beds exclude those designated for Europeans.

Nearest neighbour ratio applied for country adjustments.

All continuous variables are logged.

Standard errors in parentheses.

* *p*<0.10, ** *p*<0.05, *** *p*<0.01

## Appendix B

**Table B1.** Alternate models of scope: TME instead of TGR

	(1) Beds per capita	(2) Hospitals per capita
*Region*		
West African colony	Ref.	Ref.
East African colony	0.369***	1.106***
	(0.099)	(0.082)
Southern African colony	–1.008***	1.410***
	(0.141)	(0.134)
*European settler share*		
Non-settler colony	Ref.	Ref.
Settler colony	–0.043	–0.558***
	(0.102)	(0.100)
*Alternative providers*		
Mission hospitals per capita	–0.133***	0.029
	(0.030)	(0.031)
*Period*		
1900–19	Ref.	Ref.
1920–39	–0.167*	–0.496***
	(0.090)	(0.088)
1940–60	0.355***	–0.658***
	(0.135)	(0.135)
Real TME per capita	0.303***	0.718***
	(0.068)	(0.059)
Medical department est.	–0.275***	0.610***
	(0.073)	(0.069)
European staff per capita	0.554***	–0.031
	(0.047)	(0.059)
State hospitals per capita	0.785***	
	(0.055)	
Average no. beds per hospital		–0.227***
		(0.059)
Country area	0.000***	0.000
	(0.000)	(0.000)
Constant	6.336***	–11.668***
	(0.975)	(0.828)
Observations	305	305
r2 w	0.466	0.266
r2 b	0.971	0.899

*Note:* State hospitals and hospital beds exclude those designated for Europeans.

All country average ratio method applied for exclusions.

All continuous variables are logged.

Standard errors in parentheses.

* *p*<0.10, ** *p*<0.05, *** *p*<0.01

**Table B2.** Alternate models of utilization: TME instead of TGR

	(1) Inpatients per capita	(2) Outpatients per capita
*Region*		
West African colony	Ref.	Ref.
East African colony	0.716***	1.588***
	(0.082)	(0.127)
Southern African colony	0.767***	1.942***
	(0.128)	(0.199)
*European settler share*		
Non-settler colony	Ref.	Ref.
Settler colony	0.399***	–0.996***
	(0.081)	(0.125)
*Alternative providers*		
Mission hospitals per capita	0.164***	0.120***
	(0.025)	(0.039)
*Period*		
1900–19	Ref.	Ref.
1920–39	0.406***	0.690***
	(0.073)	(0.113)
1940–60	0.540***	0.780***
	(0.109)	(0.170)
Real TME per capita	0.280***	0.729***
	(0.057)	(0.088)
Medical department est.	0.343***	0.312***
	(0.060)	(0.094)
European staff per capita	–0.103**	–0.003
	(0.047)	(0.072)
State hospitals per capita	0.207***	–0.028
	(0.045)	(0.070)
Average no. beds per hospital	0.368***	0.174**
	(0.048)	(0.075)
Country area	–0.000	–0.000
	(0.000)	(0.000)
Constant	–4.390***	–2.357*
	(0.844)	(1.309)
Observations	296	296
r2 w	0.738	0.741
r2 b	0.987	0.948

*Note:* State hospitals and hospital beds exclude those designated for Europeans.

All country average ratio method applied for exclusions.

All continuous variables are logged.

Standard errors in parentheses.

* *p*<0.10, ** *p*<0.05, *** *p*<0.01

**Table B3.** Alternate models of scope: Mission hospitals per sq km

	(1) Beds per capita	(2) Hospitals per capita
*Region*		
West African colony	Ref.	Ref.
East African colony	0.529***	1.145***
	(0.100)	(0.085)
Southern African colony	–0.943***	1.419***
	(0.141)	(0.133)
*European settler share*		
Non-settler colony	Ref.	Ref.
Settler colony	–0.292***	–0.637***
	(0.107)	(0.106)
*Alternative providers*		
Mission hospitals per square kilometer	–0.134***	–0.103***
	(0.021)	(0.022)
*Period*		
1900–19	Ref.	Ref.
1920–39	–0.059	–0.419***
	(0.082)	(0.082)
1940–60	0.392***	–0.590***
	(0.131)	(0.133)
Real TGR per capita	0.428***	0.796***
	(0.070)	(0.061)
TME share of TGE	–0.022	0.082***
	(0.014)	(0.013)
Medical department est.	–0.219***	0.538***
	(0.074)	(0.072)
European staff per capita	0.453***	0.008
	(0.048)	(0.057)
State hospitals per capita	0.728***	
	(0.054)	
Country area	0.000***	–0.000
	(0.000)	(0.000)
Average no. beds per hospital		–0.291***
		(0.058)
Constant	4.231***	–14.596***
	(1.133)	(0.841)
Observations	304	304
r2 w	0.461	0.253
r2 b	0.980	0.925

*Note:* State hospitals and hospital beds exclude those designated for Europeans.

All country average ratio method applied for exclusions.

All continuous variables are logged.

Standard errors in parentheses.

* *p*<0.10, ** *p*<0.05, *** *p*<0.01

**Table B4.** Alternate models of utilization: Mission hospitals per sq km

	(1) Inpatients per capita	(2) Outpatients per capita
*Region*		
West African colony	Ref.	Ref.
East African colony	0.761***	1.515***
	(0.080)	(0.131)
Southern African colony	1.153***	2.207***
	(0.123)	(0.203)
*European settler share*		
Non-settler colony	Ref.	Ref.
Settler colony	0.365***	–0.806***
	(0.082)	(0.136)
*Alternative providers*		
Mission hospitals per square kilometer	0.141***	0.150***
	(0.018)	(0.030)
*Period*		
1900–19	Ref.	Ref.
1920–39	0.302***	0.665***
	(0.065)	(0.106)
1940–60	0.337***	0.639***
	(0.104)	(0.171)
Real TGR per capita	0.306***	0.507***
	(0.059)	(0.098)
TME share of TGE	–0.003	0.112***
	(0.010)	(0.017)
Medical department est.	0.482***	0.348***
	(0.058)	(0.096)
European staff per capita	–0.123***	0.046
	(0.043)	(0.071)
State hospitals per capita	0.310***	0.071
	(0.043)	(0.071)
Average no. beds per hospital	0.386***	0.314***
	(0.048)	(0.078)
Country area	0.000	–0.000
	(0.000)	(0.000)
Constant	–5.289***	–4.188***
	(0.889)	(1.465)
Observations	295	295
r2 w	0.773	0.750
r2 b	0.989	0.949

*Note:* State hospitals and state hospital beds exclude those designated for Europeans.

All country average ratio method applied for exclusions.

All continuous variables are logged.

Standard errors in parentheses.

* *p*<0.10, ** *p*<0.05, *** *p*<0.01

**Table B5.** Alternate models of scope: excluding The Gambia and Zanzibar

	(1) Beds per capita	(2) Hospitals per capita
*Region*		
West African colony	Ref.	Ref.
East African colony	0.642***	1.280***
	(0.104)	(0.090)
Southern African colony	–0.622***	1.389***
	(0.151)	(0.145)
*European settler share*		
Non-settler colony	Ref.	Ref.
Settler colony	–0.307***	–0.672***
	(0.107)	(0.110)
*Alternative providers*		
Mission hospitals per capita	–0.149***	0.023
	(0.031)	(0.035)
*Period*		
1900–19	Ref.	Ref.
1920–39	–0.117	–0.395***
	(0.082)	(0.085)
1940–60	0.245*	–0.676***
	(0.131)	(0.136)
Real TGR per capita	0.410***	0.701***
	(0.069)	(0.067)
TME share of TGE	–0.000	0.103***
	(0.014)	(0.014)
Medical department est.	–0.069	0.652***
	(0.077)	(0.072)
European staff per capita	0.518***	0.027
	(0.050)	(0.064)
State hospitals per capita	0.736***	
	(0.054)	
Country area	0.000***	0.000**
	(0.000)	(0.000)
Average no. beds per hospital		–0.307***
		(0.063)
Constant	3.491***	–13.935***
	(1.149)	(0.927)
Observations	279	279
r2 w	0.521	0.308
r2 b	0.991	0.925

*Note:* State hospitals and hospital beds exclude those designated for Europeans.

All country average ratio method applied for exclusions.

All continuous variables are logged.

Standard errors in parentheses.

* *p*<0.10, ** *p*<0.05, *** *p*<0.01

**Table B6.** Alternate models of utilization: excluding The Gambia and Zanzibar

	(1) Inpatients per capita	(2) Outpatients per capita
*Region*		
West African colony	Ref.	Ref.
East African colony	0.746***	1.407***
	(0.092)	(0.142)
Southern African colony	0.988***	2.084***
	(0.133)	(0.206)
*European settler share*		
Non-settler colony	Ref.	Ref.
Settler colony	0.321***	–0.715***
	(0.090)	(0.139)
*Alternative providers*		
Mission hospitals per capita	0.133***	0.038
	(0.029)	(0.045)
*Period*		
1900–19	Ref.	Ref.
1920–39	0.386***	0.774***
	(0.069)	(0.108)
1940–60	0.468***	0.849***
	(0.111)	(0.172)
Real TGR per capita	0.385***	0.662***
	(0.064)	(0.099)
TME share of TGE	–0.015	0.095***
	(0.012)	(0.018)
Medical department est.	0.400***	0.178*
	(0.064)	(0.099)
European staff per capita	–0.176***	0.102
	(0.052)	(0.080)
State hospitals per capita	0.249***	0.038
	(0.047)	(0.073)
Average no. beds per hospital	0.389***	0.270***
	(0.055)	(0.086)
Country area	–0.000**	–0.000**
	(0.000)	(0.000)
Constant	–5.786***	–3.945***
	(0.980)	(1.518)
Observations	270	270
r2 w	0.755	0.726
r2 b	0.995	0.979

*Note:* State hospitals and hospital beds exclude those designated for Europeans.

All country average ratio method applied for exclusions.

All continuous variables are logged.

Standard errors in parentheses.

* *p*<0.10, ** *p*<0.05, *** *p*<0.01

**Table B7.** Alternate models of scope: excluding Zanzibar

	(1) Beds per capita	(2) Hospitals per capita
*Region*		
West African colony	Ref.	Ref.
East African colony	0.528***	1.204***
	(0.107)	(0.089)
Southern African colony	–0.838***	1.383***
	(0.153)	(0.144)
*European settler share*		
Non-settler colony	Ref.	Ref.
Settler colony	–0.275**	–0.656***
	(0.113)	(0.111)
*Alternative providers*		
Mission hospitals per capita	–0.116***	0.063*
	(0.032)	(0.034)
*Period*		
1900–19	Ref.	Ref.
1920–39	–0.133	–0.417***
	(0.086)	(0.086)
1940–60	0.306**	–0.691***
	(0.138)	(0.138)
Real TGR per capita	0.364***	0.654***
	(0.071)	(0.066)
TME share of TGE	–0.014	0.099***
	(0.015)	(0.014)
Medical department est.	–0.136*	0.643***
	(0.079)	(0.073)
European staff per capita	0.466***	–0.040
	(0.051)	(0.059)
State hospitals per capita		0.793***
		(0.057)
Country area	0.000***	0.000
	(0.000)	(0.000)
Average no. beds per hospital		–0.220***
		(0.060)
Constant	4.537***	–14.265***
	(1.168)	(0.895)
Observations	292	292
r2 w	0.498	0.293
r2 b	0.971	0.898

*Note:* State hospitals and hospital beds exclude those designated for Europeans.

All country average ratio method applied for exclusions.

All continuous variables are logged.

Standard errors in parentheses.

* *p*<0.10, ** *p*<0.05, *** *p*<0.01

**Table B8.** Alternate models of utilization: excluding Zanzibar

	(1) Inpatients per capita	(2) Outpatients per capita
*Region*		
West African colony	Ref.	Ref.
East African colony	0.781***	1.368***
	(0.088)	(0.136)
Southern African colony	0.960***	2.053***
	(0.131)	(0.202)
*European settler share*		
Non-settler colony	Ref.	Ref.
Settler colony	0.306***	–0.694***
	(0.089)	(0.137)
*Alternative providers*		
Mission hospitals per capita	0.114***	0.062
	(0.027)	(0.041)
*Period*		
1900–19	Ref.	Ref.
1920–39	0.391***	0.755***
	(0.069)	(0.107)
1940–60	0.485***	0.834***
	(0.111)	(0.171)
Real TGR per capita	0.409***	0.612***
	(0.059)	(0.092)
TME share of TGE	–0.013	0.094***
	(0.011)	(0.018)
Medical department est.	0.393***	0.166*
	(0.063)	(0.097)
European staff per capita	–0.119**	0.093
	(0.046)	(0.072)
State hospitals per capita	0.229***	0.047
	(0.046)	(0.070)
Average no. beds per hospital	0.333***	0.306***
	(0.049)	(0.075)
Country area	–0.000	–0.000***
	(0.000)	(0.000)
Constant	–5.523***	–3.647**
	(0.949)	(1.465)
Observations	283	283
r2 w	0.747	0.724
r2 b	0.995	0.983

*Note:* State hospitals and hospital beds exclude those designated for Europeans.

All country average ratio method applied for exclusions.

All continuous variables are logged.

Standard errors in parentheses.

* *p*<0.10, ** *p*<0.05, *** *p*<0.01

**Table B9.** Alternate models of scope: excluding The Gambia

	(1) Beds per capita	(2) Hospitals per capita
*Region*		
West African colony	Ref.	Ref.
East African colony	0.576***	1.212***
	(0.099)	(0.089)
Southern African colony	–0.626***	1.477***
	(0.149)	(0.143)
*European settler share*		
Non-settler colony	Ref.	Ref.
Settler colony	–0.227**	–0.578***
	(0.102)	(0.107)
*Alternative providers*		
Mission hospitals per capita	–0.175***	–0.008
	(0.028)	(0.033)
*Period*		
1900–19	Ref.	Ref.
1920–39	–0.117	–0.402***
	(0.081)	(0.086)
1940–60	0.256**	–0.650***
	(0.128)	(0.136)
Real TGR per capita	0.407***	0.710***
	(0.068)	(0.067)
TME share of TGE	–0.006	0.094***
	(0.013)	(0.014)
Medical department est.	–0.118	0.613***
	(0.074)	(0.072)
European staff per capita	0.554***	0.061
	(0.048)	(0.064)
State hospitals per capita	0.765***	
	(0.053)	
Country area	0.000***	0.000
	(0.000)	(0.000)
Average no. beds per hospital		–0.282***
		(0.063)
Constant	4.161***	–13.772***
	(1.113)	(0.936)
Observations	291	291
r2 w	0.497	0.282
r2 b	0.993	0.923

*Note:* State hospitals and hospital beds exclude those designated for Europeans.

All country average ratio method applied for exclusions.

All continuous variables are logged.

Standard errors in parentheses.

* *p*<0.10, ** *p*<0.05, *** *p*<0.01

**Table B10.** Alternate models of utilization: excluding The Gambia

	(1) Inpatients per capita	(2) Outpatients per capita
*Region*		
West African colony	Ref.	Ref.
East African colony	0.810***	1.649***
	(0.086)	(0.140)
Southern African colony	0.973***	2.027***
	(0.131)	(0.212)
*European settler share*		
Non-settler colony	Ref.	Ref.
Settler colony	0.258***	–0.962***
	(0.084)	(0.137)
*Alternative providers*		
Mission hospitals per capita	0.156***	0.114***
	(0.027)	(0.043)
*Period*		
1900–19	Ref.	Ref.
1920–39	0.387***	0.778***
	(0.068)	(0.111)
1940–60	0.438***	0.771***
	(0.108)	(0.175)
Real TGR per capita	0.384***	0.677***
	(0.063)	(0.102)
TME share of TGE	–0.010	0.110***
	(0.011)	(0.018)
Medical department est.	0.434***	0.307***
	(0.061)	(0.099)
European staff per capita	–0.194***	0.040
	(0.050)	(0.082)
State hospitals per capita	0.226***	–0.049
	(0.045)	(0.073)
Average no. beds per hospital	0.375***	0.199**
	(0.054)	(0.087)
Country area	–0.000*	–0.000
	(0.000)	(0.000)
Constant	–6.125***	–5.193***
	(0.946)	(1.537)
Observations	282	282
r2 w	0.755	0.726
r2 b	0.992	0.945

*Note:* State hospitals and hospital beds exclude those designated for Europeans.

All country average ratio method applied for exclusions.

All continuous variables are logged.

Standard errors in parentheses.

* *p*<0.10, ** *p*<0.05, *** *p*<0.01

