# Supplementary material for: The expansion of colonial state healthcare in twentieth-century British Africa
Source: Med Hist. 2025 Mar 11;69(1):119–65. doi: 10.1017/mdh.2024.45 (PMC12041338; doi:10.1017/mdh.2024.45)
Supplement: Bolt and Cilliers supplementary material [file S0025727324000450sup001.pdf]

Table A1: Alternate models of scope

	(1) Beds per capita	(2) Hospitals per capita
Region		
West African colony	Ref.	Ref.
East African colony	0.436***	1.128***
	(0.102)	(0.088)
Southern African colony	-0.874***	1.484***
	(0.151)	(0.143)
European settler share		
Non-settler colony	Ref.	Ref.
Settler colony	-0.178*	-0.554***
	(0.108)	(0.109)
Alternative providers		
Mission hospitals per capita	-0.143***	0.034
	(0.029)	(0.032)
Period		
1900-1919	Ref.	Ref.
1920-1939	-0.152*	-0.426***
	(0.086)	(0.087)
1940-1960	0.299**	-0.671***
	(0.135)	(0.137)
Real TGR per capita	0.356***	0.661***
	(0.070)	(0.066)
TME share of TGE	-0.017	0.091***
	(0.014)	(0.014)
Medical department est.	-0.196**	0.608***
	(0.076)	(0.072)
European staff per capita	0.509***	-0.014
	(0.049)	(0.060)
State hospitals per capita	0.836***	
	(0.055)	
Country area	0.000***	-0.000
	(0.000)	(0.000)
Average no. beds per hospital		-0.179***
		(0.060)
Constant	5.483***	-14.226***
	(1.132)	(0.907)
Observations	304	304
r2_w	0.464	0.265
r2_b	0.976	0.898

State hospitals and hospital beds for patients of all races.
Nearest neighbour ratio applied for country adjustments.
All continuous variables are logged.
Standard errors in parentheses.
* $p<0.10$, ** $p<0.05$, *** $p<0.01$

Table A2: Alternate models of utilization

	(1) In-patients per capita	(2) Out-patients per capita
Region		
West African colony	Ref.	Ref.
East African colony	0.853***	1.613***
	(0.082)	(0.133)
Southern African colony	0.953***	1.992***
	(0.130)	(0.209)
European settler share		
Non-settler colony	Ref.	Ref.
Settler colony	0.240***	-0.942***
	(0.084)	(0.135)
Alternative providers		
Mission hospitals per capita	0.138***	0.137***
	(0.025)	(0.040)
Period		
1900-1919	0.000	0.000
	(.)	(.)
1920-1939	0.397***	0.760***
	(0.068)	(0.110)
1940-1960	0.460***	0.767***
	(0.107)	(0.173)
Real TGR per capita	0.410***	0.627***
	(0.058)	(0.094)
TME share of TGE	-0.008	0.108***
	(0.011)	(0.018)
Medical department est.	0.429***	0.290***
	(0.060)	(0.098)
European staff per capita	-0.138***	0.037
	(0.046)	(0.073)
State hospitals per capita	0.202***	
	(0.044)	
Average no. beds per hospital	0.316***	0.228***
	(0.048)	(0.077)
Country area	-0.000	-0.000*
	(0.000)	(0.000)
State hospitals per capita		-0.043
		(0.070)
Constant	-5.921***	-4.848***
	(0.927)	(1.496)
Observations	295	295
r2_w	0.745	0.724
r2_b	0.992	0.957

State hospitals and state hospital beds exclude those designated for Europeans.
Nearest neighbour ratio applied for country adjustments.
All continuous variables are logged
Standard errors in parentheses
* p<0.10, ** p<0.05, *** p<0.01

Table B1: Alternate models of scope: TME instead of TGR

	(1) Beds per capita	(2) Hospitals per capita
Region		
West African colony	Ref.	Ref.
East African colony	0.369***	1.106***
	(0.099)	(0.082)
Southern African colony	-1.008***	1.410***
	(0.141)	(0.134)
European settler share		
Non-settler colony	Ref.	Ref.
Settler colony	-0.043	-0.558***
	(0.102)	(0.100)
Alternative providers		
Mission hospitals per capita	-0.133***	0.029
	(0.030)	(0.031)
Period		
1900-1919	Ref.	Ref.
1920-1939	-0.167*	-0.496***
	(0.090)	(0.088)
1940-1960	0.355***	-0.658***
	(0.135)	(0.135)
Real TME per capita	0.303***	0.718***
	(0.068)	(0.059)
Medical department est.	-0.275***	0.610***
	(0.073)	(0.069)
European staff per capita	0.554***	-0.031
	(0.047)	(0.059)
State hospitals per capita	0.785***	
	(0.055)	
Average no. beds per hospital		-0.227***
		(0.059)
Country area	0.000***	0.000
	(0.000)	(0.000)
Constant	6.336***	-11.668***
	(0.975)	(0.828)
Observations	305	305
r2_w	0.466	0.266
r2_b	0.971	0.899

State hospitals and hospital beds exclude those designated for Europeans.
All country average ratio method applied for exclusions.
All continuous variables are logged.
Standard errors in parentheses.
* p<0.10, ** p<0.05, *** p<0.01

Table B2: Alternate models of utilization: TME instead of TGR

	(1)<br>In-patients per capita	(2)<br>Out-patients per capita
Region		
West African colony	Ref.	Ref.
East African colony	0.716***	1.588***
	(0.082)	(0.127)
Southern African colony	0.767***	1.942***
	(0.128)	(0.199)
European settler share		
Non-settler colony	Ref.	Ref.
Settler colony	0.399***	-0.996***
	(0.081)	(0.125)
Alternative providers		
Mission hospitals per capita	0.164***	0.120***
	(0.025)	(0.039)
Period		
1900-1919	Ref.	Ref.
1920-1939	0.406***	0.690***
	(0.073)	(0.113)
1940-1960	0.540***	0.780***
	(0.109)	(0.170)
Real TME per capita	0.280***	0.729***
	(0.057)	(0.088)
Medical department est.	0.343***	0.312***
	(0.060)	(0.094)
European staff per capita	-0.103**	-0.003
	(0.047)	(0.072)
State hospitals per capita	0.207***	-0.028
	(0.045)	(0.070)
Average no. beds per hospital	0.368***	0.174**
	(0.048)	(0.075)
Country area	-0.000	-0.000
	(0.000)	(0.000)
Constant	-4.390***	-2.357*
	(0.844)	(1.309)
Observations	296	296
r2_w	0.738	0.741
r2_b	0.987	0.948

State hospitals and hospital beds exclude those designated for Europeans.
All country average ratio method applied for exclusions.
All continuous variables are logged.
Standard errors in parentheses.
* $p<0.10$, ** $p<0.05$, *** $p<0.01$

Table B3: Alternate models of scope: Mission hospitals per sqkm

	(1)<br>Beds per capita	(2)<br>Hospitals per capita
Region		
West African colony	Ref.	Ref.
East African colony	0.529***	1.145***
	(0.100)	(0.085)
Southern African colony	-0.943***	1.419***
	(0.141)	(0.133)
European settler share		
Non-settler colony	Ref.	Ref.
Settler colony	-0.292***	-0.637***
	(0.107)	(0.106)
Alternative providers		
Mission hospitals per square kilometer	-0.134***	-0.103***
	(0.021)	(0.022)
Period		
1900-1919	Ref.	Ref.
1920-1939	-0.059	-0.419***
	(0.082)	(0.082)
1940-1960	0.392***	-0.590***
	(0.131)	(0.133)
Real TGR per capita	0.428***	0.796***
	(0.070)	(0.061)
TME share of TGE	-0.022	0.082***
	(0.014)	(0.013)
Medical department est.	-0.219***	0.538***
	(0.074)	(0.072)
European staff per capita	0.453***	0.008
	(0.048)	(0.057)
State hospitals per capita	0.728***	
	(0.054)	
Country area	0.000***	-0.000
	(0.000)	(0.000)
Average no. beds per hospital		-0.291***
		(0.058)
Constant	4.231***	-14.596***
	(1.133)	(0.841)
Observations	304	304
r2_w	0.461	0.253
r2_b	0.980	0.925

State hospitals and hospital beds exclude those designated for Europeans.
All country average ratio method applied for exclusions.
All continuous variables are logged.
Standard errors in parentheses.
* p<0.10, ** p<0.05, *** p<0.01

Table B4: Alternate models of utilization: Mission hospitals per sqkm

	(1) In-patients per capita	(2) Out-patients per capita
Region		
West African colony	Ref.	Ref.
East African colony	0.761***	1.515***
	(0.080)	(0.131)
Southern African colony	1.153***	2.207***
	(0.123)	(0.203)
European settler share		
Non-settler colony	Ref.	Ref.
Settler colony	0.365***	-0.806***
	(0.082)	(0.136)
Alternative providers		
Mission hospitals per square kilometer	0.141***	0.150***
	(0.018)	(0.030)
Period		
1900-1919	Ref.	Ref.
1920-1939	0.302***	0.665***
	(0.065)	(0.106)
1940-1960	0.337***	0.639***
	(0.104)	(0.171)
Real TGR per capita	0.306***	0.507***
	(0.059)	(0.098)
TME share of TGE	-0.003	0.112***
	(0.010)	(0.017)
Medical department est.	0.482***	0.348***
	(0.058)	(0.096)
European staff per capita	-0.123***	0.046
	(0.043)	(0.071)
State hospitals per capita	0.310***	0.071
	(0.043)	(0.071)
Average no. beds per hospital	0.386***	0.314***
	(0.048)	(0.078)
Country area	0.000	-0.000
	(0.000)	(0.000)
Constant	-5.289***	-4.188***
	(0.889)	(1.465)
Observations	295	295
r2_w	0.773	0.750
r2_b	0.989	0.949

State hospitals and state hospital beds exclude those designated for Europeans.
All country average ratio method applied for exclusions.
All continuous variables are logged.
Standard errors in parentheses.
* p<0.10, ** p<0.05, *** p<0.01

Table B5: Alternate models of scope: excluding the Gambia and Zanzibar

	(1) Beds per capita	(2) Hospitals per capita
Region		
West African colony	Ref.	Ref.
East African colony	0.642***	1.280***
	(0.104)	(0.090)
Southern African colony	-0.622***	1.389***
	(0.151)	(0.145)
European settler share		
Non-settler colony	Ref.	Ref.
Settler colony	-0.307***	-0.672***
	(0.107)	(0.110)
Alternative providers		
Mission hospitals per capita	-0.149***	0.023
	(0.031)	(0.035)
Period		
1900-1919	Ref.	Ref.
1920-1939	-0.117	-0.395***
	(0.082)	(0.085)
1940-1960	0.245*	-0.676***
	(0.131)	(0.136)
Real TGR per capita	0.410***	0.701***
	(0.069)	(0.067)
TME share of TGE	-0.000	0.103***
	(0.014)	(0.014)
Medical department est.	-0.069	0.652***
	(0.077)	(0.072)
European staff per capita	0.518***	0.027
	(0.050)	(0.064)
State hospitals per capita	0.736***	
	(0.054)	
Country area	0.000***	0.000**
	(0.000)	(0.000)
Average no. beds per hospital		-0.307***
		(0.063)
Constant	3.491***	-13.935***
	(1.149)	(0.927)
Observations	279	279
r2_w	0.521	0.308
r2_b	0.991	0.925

State hospitals and hospital beds exclude those designated for Europeans.
All country average ratio method applied for exclusions.
All continuous variables are logged.
Standard errors in parentheses.
* p<0.10, ** p<0.05, *** p<0.01

Table B6: Alternate models of utilization: excluding the Gambia and Zanzibar

	(1) In-patients per capita	(2) Out-patients per capita
Region		
West African colony	Ref.	Ref.
East African colony	0.746***	1.407***
	(0.092)	(0.142)
Southern African colony	0.988***	2.084***
	(0.133)	(0.206)
European settler share		
Non-settler colony	Ref.	Ref.
Settler colony	0.321***	-0.715***
	(0.090)	(0.139)
Alternative providers		
Mission hospitals per capita	0.133***	0.038
	(0.029)	(0.045)
Period		
1900-1919	Ref.	Ref.
1920-1939	0.386***	0.774***
	(0.069)	(0.108)
1940-1960	0.468***	0.849***
	(0.111)	(0.172)
Real TGR per capita	0.385***	0.662***
	(0.064)	(0.099)
TME share of TGE	-0.015	0.095***
	(0.012)	(0.018)
Medical department est.	0.400***	0.178*
	(0.064)	(0.099)
European staff per capita	-0.176***	0.102
	(0.052)	(0.080)
State hospitals per capita	0.249***	0.038
	(0.047)	(0.073)
Average no. beds per hospital	0.389***	0.270***
	(0.055)	(0.086)
Country area	-0.000**	-0.000**
	(0.000)	(0.000)
Constant	-5.786***	-3.945***
	(0.980)	(1.518)
Observations	270	270
r2_w	0.755	0.726
r2_b	0.995	0.979

State hospitals and hospital beds exclude those designated for Europeans.
All country average ratio method applied for exclusions.
All continuous variables are logged.
Standard errors in parentheses.
* p<0.10, ** p<0.05, *** p<0.01

Table B7: Alternate models of scope: excluding Zanzibar

	(1) Beds per capita	(2) Hospitals per capita
Region		
West African colony	Ref.	Ref.
East African colony	0.528***	1.204***
	(0.107)	(0.089)
Southern African colony	-0.838***	1.383***
	(0.153)	(0.144)
European settler share		
Non-settler colony	Ref.	Ref.
Settler colony	-0.275**	-0.656***
	(0.113)	(0.111)
Alternative providers		
Mission hospitals per capita	-0.116***	0.063*
	(0.032)	(0.034)
Period		
1900-1919	Ref.	Ref.
1920-1939	-0.133	-0.417***
	(0.086)	(0.086)
1940-1960	0.306**	-0.691***
	(0.138)	(0.138)
Real TGR per capita	0.364***	0.654***
	(0.071)	(0.066)
TME share of TGE	-0.014	0.099***
	(0.015)	(0.014)
Medical department est.	-0.136*	0.643***
	(0.079)	(0.073)
European staff per capita	0.466***	-0.040
	(0.051)	(0.059)
State hospitals per capita	0.793***	
	(0.057)	
Country area	0.000***	0.000
	(0.000)	(0.000)
Average no. beds per hospital		-0.220***
		(0.060)
Constant	4.537***	-14.265***
	(1.168)	(0.895)
Observations	292	292
r2_w	0.498	0.293
r2_b	0.971	0.898

State hospitals and hospital beds exclude those designated for Europeans.
All country average ratio method applied for exclusions.
All continuous variables are logged.
Standard errors in parentheses.
* $p<0.10$, ** $p<0.05$, *** $p<0.01$

Table B8: Alternate models of utilization: excluding Zanzibar

	(1) In-patients per capita	(2) Out-patients per capita
Region		
West African colony	Ref.	Ref.
East African colony	0.781***	1.368***
	(0.088)	(0.136)
Southern African colony	0.960***	2.053***
	(0.131)	(0.202)
European settler share		
Non-settler colony	Ref.	Ref.
Settler colony	0.306***	-0.694***
	(0.089)	(0.137)
Alternative providers		
Mission hospitals per capita	0.114***	0.062
	(0.027)	(0.041)
Period		
1900-1919	Ref.	Ref.
1920-1939	0.391***	0.755***
	(0.069)	(0.107)
1940-1960	0.485***	0.834***
	(0.111)	(0.171)
Real TGR per capita	0.409***	0.612***
	(0.059)	(0.092)
TME share of TGE	-0.013	0.094***
	(0.011)	(0.018)
Medical department est.	0.393***	0.166*
	(0.063)	(0.097)
European staff per capita	-0.119**	0.093
	(0.046)	(0.072)
State hospitals per capita	0.229***	0.047
	(0.046)	(0.070)
Average no. beds per hospital	0.333***	0.306***
	(0.049)	(0.075)
Country area	-0.000	-0.000***
	(0.000)	(0.000)
Constant	-5.523***	-3.647**
	(0.949)	(1.465)
Observations	283	283
r2_w	0.747	0.724
r2_b	0.995	0.983

State hospitals and hospital beds exclude those designated for Europeans.
All country average ratio method applied for exclusions.
All continuous variables are logged.
Standard errors in parentheses.
* p<0.10, ** p<0.05, *** p<0.01

Table B9: Alternate models of scope: excluding the Gambia

	(1)<br>Beds per capita	(2)<br>Hospitals per capita
Region		
West African colony	Ref.	Ref.
East African colony	0.576***	1.212***
	(0.099)	(0.089)
Southern African colony	-0.626***	1.477***
	(0.149)	(0.143)
European settler share		
Non-settler colony	Ref.	Ref.
Settler colony	-0.227**	-0.578***
	(0.102)	(0.107)
Alternative providers		
Mission hospitals per capita	-0.175***	-0.008
	(0.028)	(0.033)
Period		
1900-1919	Ref.	Ref.
1920-1939	-0.117	-0.402***
	(0.081)	(0.086)
1940-1960	0.256**	-0.650***
	(0.128)	(0.136)
Real TGR per capita	0.407***	0.710***
	(0.068)	(0.067)
TME share of TGE	-0.006	0.094***
	(0.013)	(0.014)
Medical department est.	-0.118	0.613***
	(0.074)	(0.072)
European staff per capita	0.554***	0.061
	(0.048)	(0.064)
State hospitals per capita	0.765***	
	(0.053)	
Country area	0.000***	0.000
	(0.000)	(0.000)
Average no. beds per hospital		-0.282***
		(0.063)
Constant	4.161***	-13.772***
	(1.113)	(0.936)
Observations	291	291
r2_w	0.497	0.282
r2_b	0.993	0.923

State hospitals and hospital beds exclude those designated for Europeans.
All country average ratio method applied for exclusions.
All continuous variables are logged.
Standard errors in parentheses.
* p<0.10, ** p<0.05, *** p<0.01

Table B10: Alternate models of utilization: excluding the Gambia

	(1) In-patients per capita	(2) Out-patients per capita
Region		
West African colony	Ref.	Ref.
East African colony	0.810***	1.649***
	(0.086)	(0.140)
Southern African colony	0.973***	2.027***
	(0.131)	(0.212)
European settler share		
Non-settler colony	Ref.	Ref.
Settler colony	0.258***	-0.962***
	(0.084)	(0.137)
Alternative providers		
Mission hospitals per capita	0.156***	0.114***
	(0.027)	(0.043)
Period		
1900-1919	Ref.	Ref.
1920-1939	0.387***	0.778***
	(0.068)	(0.111)
1940-1960	0.438***	0.771***
	(0.108)	(0.175)
Real TGR per capita	0.384***	0.677***
	(0.063)	(0.102)
TME share of TGE	-0.010	0.110***
	(0.011)	(0.018)
Medical department est.	0.434***	0.307***
	(0.061)	(0.099)
European staff per capita	-0.194***	0.040
	(0.050)	(0.082)
State hospitals per capita	0.226***	-0.049
	(0.045)	(0.073)
Average no. beds per hospital	0.375***	0.199**
	(0.054)	(0.087)
Country area	-0.000*	-0.000
	(0.000)	(0.000)
Constant	-6.125***	-5.193***
	(0.946)	(1.537)
Observations	282	282
r2_w	0.755	0.726
r2_b	0.992	0.945

State hospitals and hospital beds exclude those designated for Europeans.
All country average ratio method applied for exclusions.
All continuous variables are logged.
Standard errors in parentheses.
* p<0.10, ** p<0.05, *** p<0.01
